# Imaging abnormalities of the acromioclavicular joint and subacromial space are common in asymptomatic shoulders: a systematic review

**DOI:** 10.1186/s13018-024-05378-4

**Published:** 2025-01-03

**Authors:** Thomas Ibounig, Lasse Rämö, Romi Haas, Mark Jones, Teppo L. N. Järvinen, Simo Taimela, Sean Docking, Sharon Sanders, Rachelle Buchbinder

**Affiliations:** 1 Finnish Centre for Evidence-Based Orthopaedics, University of Helsinki, Helsinki, Finland; 2 Department of Orthopaedics and Traumatology, Helsinki University Hospital, Haartmaninkatu 4, Building 4, P.O. Box 320, 00029 Helsinki, Finland; 3 School of Public Health and Preventive Medicine, Musculoskeletal Health and Wiser Health Care Units, Monash University, Melbourne, VIC Australia; 4 Institute for Evidence-Based Healthcare, Faculty of Health Sciences and Medicine, Bond University, Gold Coast, Australia

**Keywords:** Shoulder, Imaging abnormalities, Prevalence, Asymptomatic, Acromioclavicular joint, Subacromial space, Systematic review

## Abstract

**Objectives:**

To determine the prevalence of acromioclavicular (AC) joint and subacromial space imaging abnormalities in asymptomatic adults, with a secondary objective of comparing findings between asymptomatic and symptomatic shoulders within the same study populations.

**Methods:**

We conducted a systematic review of studies examining shoulder imaging abnormalities detected by X-ray, ultrasound (US), computed tomography (CT), and magnetic resonance imaging (MRI) in asymptomatic adults (PROSPERO registration CRD42018090041). This report focuses on AC joint and subacromial space abnormalities. Databases searched included Ovid MEDLINE, Embase, CINAHL and Web of Science from inception to June 2023. Our primary analysis used data from population-based studies, and risk of bias and certainty of evidence were evaluated with tools for prognostic studies.

**Results:**

Thirty-one studies (4 X-ray, 11 US, 15 MRI, 1 both X-ray and MRI) provided useable prevalence data. One study was population-based (20 shoulders), 16 included miscellaneous study populations (2436 shoulders) and 14 focused on athletes (708 shoulders). The certainty of evidence was very low mainly due to high risk of bias, imprecision, and indirectness across studies. Clinical diversity precluded pooling. Population-based prevalence of acromioclavicular osteoarthritis (AC OA) ranged from 85% on MRI to 95% on X-ray. In other study populations, AC OA prevalence in asymptomatic shoulders varied from 6 to 47% on X-ray, 1 to 65% on US, and 0 to 82% on MRI. Among eight studies that included both asymptomatic and symptomatic shoulders, AC OA prevalence ranged from 13 to 95% in asymptomatic shoulders and from 20 to 100% in symptomatic shoulders.

**Conclusion:**

The prevalence of AC joint and subacromial space abnormalities in asymptomatic shoulders is highly variable, and often comparable to that in symptomatic shoulders. Due to the low certainty of evidence and significant variation among study populations, further research is needed to clarify these prevalence estimates and to guide evidence-based management of shoulder abnormalities.

**Supplementary Information:**

The online version contains supplementary material available at 10.1186/s13018-024-05378-4.

## Key message

- Structural abnormalities in shoulder imaging are frequently observed in asymptomatic adults, raising concerns about their clinical significance
- Careful interpretation of imaging results is crucial to avoid unnecessary interventions based solely on imaging findings.
- More accurate prevalence data are essential for developing evidence-based approaches to managing shoulder pain in clinical practice.

**Supplementary Information:**

The online version contains supplementary material available at 10.1186/s13018-024-05378-4.

## Introduction

Shoulder symptoms are a prevalent source of musculoskeletal pain and disability, affecting approximately one-quarter of the population [1]. Shoulder imaging is frequently used to complement clinical examination [2, 3] and may detect abnormalities such as degenerative and traumatic rotator cuff injuries, labral and biceps pathology, glenohumeral and acromioclavicular joint arthritis (AC OA), subacromial bursal enlargement or inflammation, and fractures, most commonly fractures of the humeral head or clavicle [4, 5]. While it seems logical to associate these structural abnormalities with symptoms and to consider surgical correction if symptoms persist, many of these abnormalities are also commonly observed in asymptomatic individuals, particularly in the aging population [6–8].

Imaging modalities such as X-ray, ultrasound (US), computed tomography (CT), and magnetic resonance imaging (MRI) have distinct strengths and limitations in identifying structural abnormalities. X-rays are cost-effective for bony structures but lack soft tissue assessment, while US provides dynamic imaging of soft tissues, depending on operator skill. CT excels in detailed bony anatomy visualization but has higher radiation exposure and limited soft tissue utility. MRI, the gold standard, effectively evaluates both soft tissue and bone but is costly and less accessible.

The overall aim of the The SystematiC Review of shoUlder imaging abnormaliTies IN asYmptomatic adults (SCRUTINY) study was to summarize the prevalence of shoulder imaging abnormalities in asymptomatic adults. The primary objective of this paper was to assess the prevalence of abnormalities of the acromioclavicular (AC) joint and subacromial (SA) space from (a) population-based studies, and (b) other study populations, such as volunteers, healthcare-populations, and athletes. Our secondary objective was to compare the prevalence of imaging abnormalities in adults with and without symptoms from the same or comparable study populations.

## Methods

The SCRUTINY systematic review adheres to the Preferred Reporting Items for Systematic reviews and Meta-Analysis (PRISMA) 2020 Statement and is registered with PROSPERO (CRD42018090041) [9]. This paper presents findings related to abnormalities of the AC joint and SA space. Part I of the SCRUTINY study details the findings concerning the glenohumeral joint, while Part III focuses on rotator cuff abnormalities.

### Inclusion criteria

Observational population-based studies with asymptomatic adult participants (18 years and older) reporting on the prevalence of (i) AC OA, (ii) SA bursal abnormalities, (iii) SA space abnormalities, and (iv) SA calcification, as detected by X-ray, ultrasound (US), computed tomography (CT) and magnetic resonance imaging (MRI), were included.

Given the limited number of population-based studies—those conducted in general populations rather than recruiting from specific groups like athletes or individuals with particular characteristics—we also included research involving other groups, such as community volunteers, healthcare populations and athletes. Studies that reported on both asymptomatic and symptomatic shoulders, whether from the same individuals or different individuals within the same study population were also included. Detailed eligibility criteria are provided in Supplementary Table 1.

### Search strategy

We conducted a comprehensive search of Ovid MEDLINE, Embase, CINAHL, and Web of Science from their inception up to June 12, 2023, without imposing language restrictions. The search strings used for each database are detailed in Supplementary Table 2. Additionally, on June 16, 2023, we performed a backward and forward citation analysis of the included studies using Scopus.

### Study selection and screening

The titles and abstracts of identified studies were independently screened by five authors (SLS, RH, RJ, TI, and LR). Full-text papers of potentially eligible studies were then retrieved and thoroughly reviewed to determine their eligibility. Disagreements were resolved by a third author (RB or TI) in cases where consensus could not be achieved. Reasons for the exclusion of ineligible studies were documented.

### Assessment of risk of bias

Pairs of reviewers (SLS & RH or TI & LR) independently evaluated each study using a modified version of the risk of bias assessment tool originally developed by Hoy et al. [10]. This adapted version comprised seven items targeting essential domains for assessing the risk of bias in prevalence studies, mainly regarding selection bias and measurement bias. An overall judgment of the risk of bias was assigned as high, moderate, or low. Detailed information regarding the adaptations and guidance for conducting the risk of bias assessment can be found in Supplementary Table 3.

### Data extraction

Using a pre-tested data extraction template, we extracted study details, participant demographics (population-based, athletes, or miscellaneous populations including community volunteers and healthcare populations), imaging modalities (X-ray, US, CT, or MRI), and prevalence findings (AC OA, SA bursa, SA space, SA calcification). In instances where studies conducted shoulder imaging but did not provide prevalence data categorized by shoulder symptom status, we contacted the first and last study authors via email to request this information.

### Data analysis and synthesis

Given that most of the included studies presented prevalence data of AC joint and SA space abnormalities per shoulder and not per individual, we chose to analyze the data based on the number of shoulders rather than number of participants. Prevalence estimates and their corresponding 95% confidence intervals were calculated using the Freeman-Tukey double arcsine transformation and exact confidence intervals, with each calculation based on one shoulder per individual. Initially, our primary analysis was aimed at the general population.

Due to clinical heterogeneity of the included studies, it was inappropriate to perform meta-analyses. We therefore conducted a narrative synthesis of the studies reporting the prevalence of imaging abnormalities in asymptomatic shoulders.

We also performed a narrative synthesis of studies reporting the prevalence of structural abnormalities in both asymptomatic and symptomatic shoulders from the same individuals or study populations. However, studies comparing the prevalence of imaging abnormalities in asymptomatic individuals with a different group of participants experiencing symptoms (for example, comparing symptomatic athletes with asymptomatic non-athletes) were excluded from this analysis.

### Patient and public involvement

Patients and the general public did not participate in the planning or conduct of this systematic review.

### Certainty of the evidence

Currently there is no specific Grading of Recommendations, Assessment, Development, and Evaluation (GRADE) framework tailored for prevalence studies. Consequently, we adapted the GRADE approach for prognostic studies, as described by Iorio et al. [11] (Supplementary Table 4). We evaluated the certainty of evidence independently for each outcome and study population.

### Deviations from the protocol

Deviations from the planned methods, along with their rationale, are outlined in detail in Supplementary Table 5.

## Results

### Search results

**Fig. 1:**
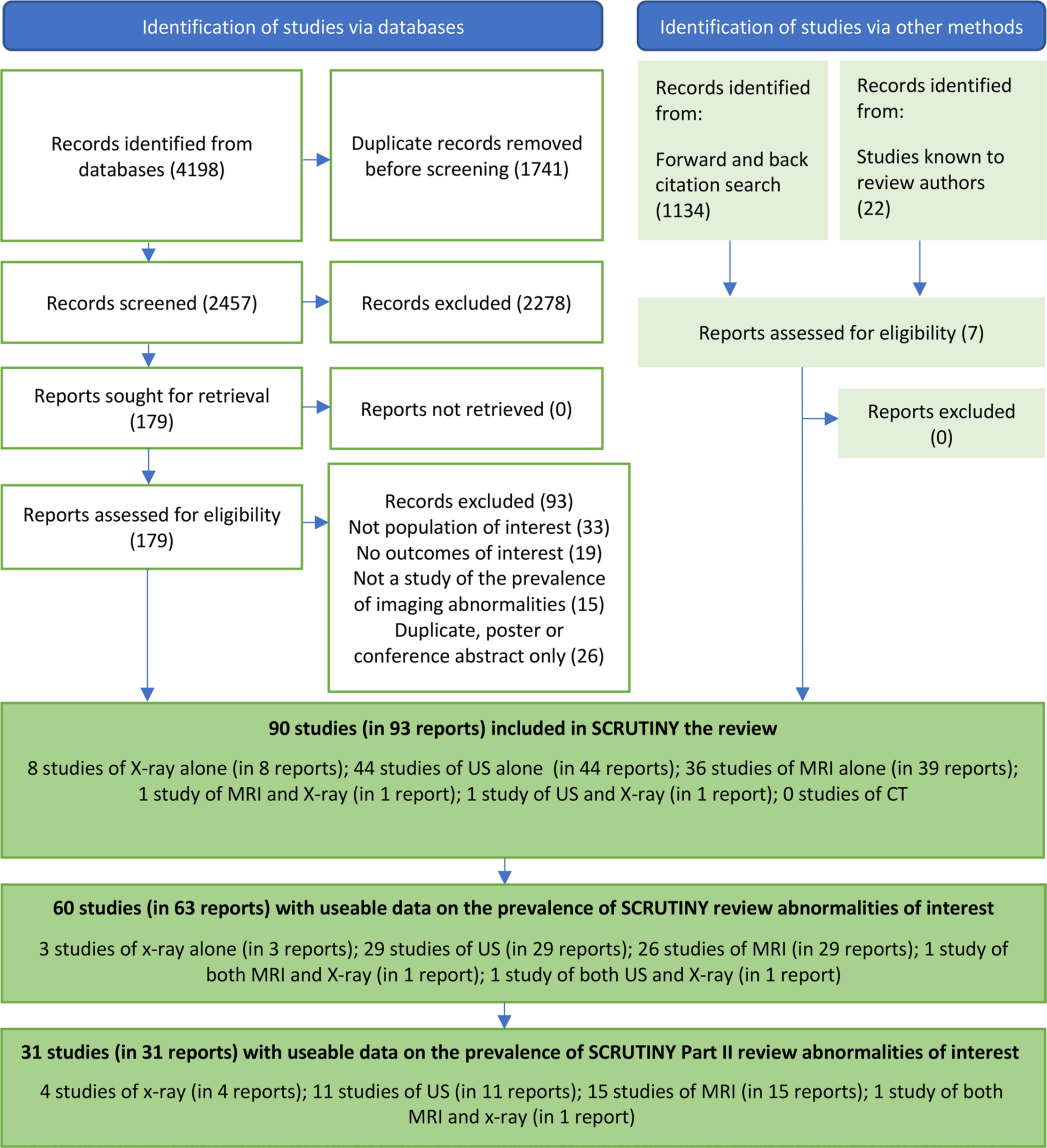
PRISMA flow diagram showing search and screening results. Abbreviations: US, ultrasound; MRI, magnetic resonance imaging; SCRUTINY, the SystematiC Review of shoUlder imaging abnormaliTies IN asYmptomatic adults

A total of 2457 records were identified through database searches, with an additional 1156 records obtained via other methods (Fig. 1). Following a full-text review of 186 papers, 93 studies were excluded. The reasons for exclusion are detailed in Supplementary Table 6. Studies that met the eligibility criteria but did not provide usable prevalence data are listed in Supplementary Table 7.

Overall, the SCRUTINY review included 90 studies reported in 93 publications. In this paper, 31 studies with usable data were included, comprising 4 X-ray [12–15], 11 US [6, 16–25], 15 MRI [5, 7, 8, 26–37], and 1 including both X-ray and MRI [4]. No CT studies were found. There was one population-based study (*n* = 20 shoulders) [4], 16 studies with miscellaneous populations (including volunteers [5, 7, 12, 22, 26, 27, 29], healthcare populations [6, 8, 17, 18, 20, 24, 38], a mixed population of volunteers and athletes [16], or a combination of volunteers and healthcare populations [14]) (*n* = 2436 shoulders), and 14 studies reporting on athletes (*n* = 708 shoulders) [13, 15, 19, 23, 25, 28, 31–37, 39]. Among the athlete studies, four also included volunteers [13, 19, 23, 28], with data presented separately for each group.

### Study characteristics

Table 1 summarizes the characteristics of 31 studies reporting shoulder prevalence data across population-based, miscellaneous, and athletic groups. The single population-based study involved a longitudinal cohort of 4056 adults, with imaging findings from 30 participants aged 65 years on average, predominantly female (60%) [4].

**Table 1:** Prevalence of acromioclavicular (AC) joint osteoarthritis (OA), subacromial (SA) bursa abnormality, SA space abnormality, and SA calcification in asymptomatic shoulders of population-based, miscellaneous, and athlete populations according to imaging modality (X-ray, ultrasound, and magnetic resonance imaging [MRI])

Study	Study population	Study location	Mean age, years (range)	Women, %	No of participants (No of shoulders)	AC-joint OA*, % (n/N)	SA bursa abnormality†, % (n/N)	SA space abnormality‡, % (n/N)	SA calcification, % (n/N)
**X-ray and MRI studies**									
Gill et al. 2014	Population-based X-ray MRI	Australia	64.8 (56–74)	60	20 (20)	95 (19/20) 85 (17/20)	90 (18/20)	20 (4/20)	5 (1/20)
**X-ray studies**									
Worland et al. 2003	Miscellaneous (volunteers)	USA	60.2 45 (40–49) 54 (50–59) 65 (60–69) 77 (70–)	50.8	59 (118) 15 (30) 15 (30) 14 (28) 15 (30)	NR	NR	42.4 (50/118) 20 (6/30) 56.7 (17/30) 25 (7/28) 66.7 (20/30)	NR
Maquirriain et al. 2006	Miscellaneous (volunteers)	Argentina	59.8 (51–76)	6	18 (36)	19.4 (7/36)	NR	NR	0 (0/36)
Khoschnau et al. 2020	Miscellaneous (healthcare population and volunteers)	Sweden	66 (50–75)	51	106 (129)	31 (40/129)	NR	NR	NR
Maquirriain et al. 2006	Athletes (former elite tennis players)	Argentina	57.2 (51–75)	6	18 (36)	41.7 (15/36)	NR	NR	2.8 (1/36)
Wright et al. 2007	Athletes (overhead, baseball pitchers)	USA	29 (19–43)	0	57 (57)	47.4 (27/57)	NR	NR	NR
**Ultrasound studies**									
Wang et al. 2005	Miscellaneous (volunteers and athletes)	Taiwan	21	NR	28 (56)	12.5 (7/56)	NR	NR	NR
Oschman et al. 2007	Miscellaneous (healthcare population, contralateral rotator cuff tear)	South Africa	64 (40–83)	36	50 (50)	NR	78 (39/50)	86 (43/50)	NR
Abate et al. 2010	Miscellaneous (healthcare population, with and without diabetes)	Italy	71 (65–84)	38	80 (160)	NR	18.8 (30/160)	NR	NR
Ocguder et al. 2010	Miscellaneous (asymptomatic volunteers)	Turkey	25 (18–33)	30	43 (86)	NR	0 (0/86)	NR	0 (0/86)
Girish et al. 2011	Miscellaneous (healthcare population, males with knee problems)	USA	56 (40–70)	0	51 (51)	64.7 (33/51)	78.4 (40/51)	5.9 (3/51)	3.9 (2/51)
Iagnocco et al. 2013	Miscellaneous (healthy volunteers from four rheumatologic units)	Italy	44.2 (20–85) 26 (20–29) 34 (30–39) 44 (40–49) 55 (50–59) 66 (60–)	54	97 (194) 21 (42) 19 (38) 20 (40) 19 (38) 18 (36)	25.7 (50/194) 7.1 (3/42) 7.9 (3/38) 17.5 (7/40) 47.4 (18/38) 52.8 (19/36)	11.3 (22/194) 0 (0/42) 7.9 (3/38) 15 (6/40) 15.8 (6/38) 19.4 (7/36)	2.6 (5/194) 0 (0/42) 0 (0/38) 0 (0/40) 10.5 (4/38) 2.7 (1/36)	18 (35/194) 2.4 (1/42) 10.5 (4/38) 25 (10/40) 5.3 (2/38) 50 (18/36)
Sansone et al. 2016	Miscellaneous (healthcare population, females referred to routine gynecological screening)	Italy	38.5 (18–60)	100	(509)	NR	NR	NR	13.6 (69/509)
Meroni et al. 2017	Miscellaneous (volunteers, working aged women)	Italy	36.7 (19–56)	100	228 (456)	0.9 (4/456)	0.2 (1/456)	0 (0/456)	5.7 (26/456)
Suzuki et al. 2021	Miscellaneous (volunteers)	Japan	51.2 (33–65)	60	20 (40)	NR	2.5 (1/40)	NR	7.5 (3/40)
Eliason et al. 2022	Miscellaneous (healthcare population, primary healthcare patients with unilateral shoulder pain)	Sweden	45.0 (20–59) (20–29) (30–39) (40–49) (50–59)	53	115 (115) 14 (14) 19 (19) 35 (35) 47 (47)	13 (15/115) 0 (0/14) 21.1 (4/19) 17.1 (6/35) 10.6 (5/47)	73 (84/115) 35.7 (5/14) 89.5 (17/19) 71.4 (25/35) 78.7 (37/47)	NR	17.4 (20/115) 0 (0/14) 10.5 (2/19) 20 (7/35) 23.4 (11/47)
Brasseur et al. 2004	Athletes (veteran tennis players)	France	55 (37–77)	43	119 (119)	NR	22.7 (27/119)	NR	25.2 (30/119)
Ocguder et al. 2010	Athletes (overhead sports)	Turkey	22 (17–40)	18	45 (90)	NR	20 (18/90)	NR	2.2 (2/90)
Suzuki et al. 2021	Athletes (masters level swimmers)	Japan	51.8 (33–65)	60	40 (60)	NR	11.7 (7/60)	NR	16.7 (10/60)

Study	Study population	Study location	Mean age, years (range)	Women, %	No of participants (No of shoulders)	AC-joint OA*, % (n/N)	SA bursa abnormality†, % (n/N)	SA space abnormality‡, % (n/N)	SA calcification, % (n/N)
**MRI studies**									
Chandnani et al. 1992	Miscellaneous (volunteers)	USA	(25–55)	NR	20 (20)	35 (7/20)	0 (0/20)	NR	NR
Neumann et al. 1992	Miscellaneous (volunteers)	USA	26 (22–45)	28	55 (32)	43.6 (24/55)	20 (11/55)	3.6 (2/55)	NR
Needell et al. 1996	Miscellaneous (volunteers without shoulder pain participating in a sports medicine study)	USA	54 (19–88) 29 (19–39) 50 (40–60) 75 (61–88)	51	100 (100) 26 (26) 26 (26) 48 (48)	76 (76/100) 38.5 (10/26) 88.5 (23/26) 89.6 (43/48)	33 (33/100) 19.2 (5/26) 19.2 (5/26) 47.9 (23/48)	39 (39/100) 15.4 (4/26) 26.9 (7/26) 58.3 (28/48)	NR
Stein et al. 2001	Miscellaneous (healthcare population, other musculoskeletal complaint)	USA	35 (19–72) 25 (19–30) 42 (31–72)	57	42 (50) (19)(31)	82 (41/50) 68.4 (13/19)90.3 (28/31)	NR	NR	NR
Barreto et al. 2019	Miscellaneous (volunteers with unilateral shoulder pain from the community)	Brazil	39.4 (18–77)	46	123 (123)	73.2 (90/123)	52.8 (65/123)	13 (16/123)	NR
Su et al. 2020	Miscellaneous (male volunteers from the study institution)	Taiwan	25.3 (22–29)	0	30 (30)	NR	13.3 (4/30)	NR	NR
Liu et al. 2021	Miscellaneous (volunteers, healthy non-athletic young adults)	USA	24 (20–29)	66	29 (58)	0 (0/58)	0 (0/58)	1.7 (1/58)	NR
Miniaci et al. 2002	Athletes (professional male baseball pitchers)	Canada	20.1 (18–22)	0	14 (28)	35.7 (10/28)	78.6 (22/28)	46.4 (13/28)	NR
Connor et al. 2003	Athletes (elite overhead athletes)	USA	26.4 (18–38)	NR	20 (40)	NR	47.5 (19/40)	NR	NR
Reuter et al. 2008	Athletes (Ironman participants)	USA	35 (29–62)	29	7 (7)	71.4 (5/7)	NR	NR	NR
Del Grande et al. 2016	Athletes (male overhead athletes)	USA	19.9 (17–22)	0	19 (19)	21.1 (4/19)	63.2 (12/19)	NR	NR
Celliers et al. 2017	Athletes (elite swimmers)	South Africa	18.9 (16–25)	45	20 (29)	34.5 (10/29)	34.5 (10/29)	NR	NR
Hacken et al. 2019	Athletes (college and professional male ice hockey players)	USA	22.1 (18–28)	0	25 (49)	8.2 (4/49)	NR	NR	NR
Lee et al. 2020	Athletes (elite volleyball players)	USA	25.5 (21–30)	46	26 (26)	69.2 (18/26)	NR	NR	NR
Su et al. 2020	Athletes (male baseball players)	Taiwan	25.6 (18–35)	0	68 (68)	NR	55.9 (38/68)	NR	NR
Cooper et al. 2022	Athletes (elite rock climbers)	USA	34.1 (20–60)	42	50 (100)	28 (28/100)	79 (79/100)	NR	NR

* = Osteophytes, joint effusion, bone oedema, bony ridging, elevated bone marrow signal, joint narrowing, joint degeneration, joint hypertrophy, articular surface irregularity, articular cartilage thinning, fissuring or degeneration, cortical irregularities, margin irregularity, bone sclerosis, erosions, osteoarthritis, synovial scarring, cystic change

† = Bursal effusion, bursal thickening, bursal hypertrophy

‡ = SA space narrowing, SA spurs, SA enthesophytes, acromion osteophytes, acromiohumeral distance (abnormal/narrow), Type III acromion (hooked), SA impingement, AC joint osteophytes impinging the supraspinatus tendon

Sixteen studies focused on miscellaneous populations, with a wide range of participants in terms of age (21–71 years) and sex distribution (0–100% female). These included asymptomatic volunteers [6, 7, 12, 16, 22, 26, 27, 29], individuals with unrelated health conditions [8, 14, 18, 20, 21], and those with contralateral shoulder symptoms [5, 17, 24].

Fourteen studies focused on athletic populations, including 13 on overhead sports [13, 15, 19, 23, 25, 28, 31–34, 36, 37, 39] and one on ice hockey players [35], with participant ages ranging from 19 to 57 years and female representation varying between 0 and 60%.

### Risk of bias of included studies

**Fig. 2:**
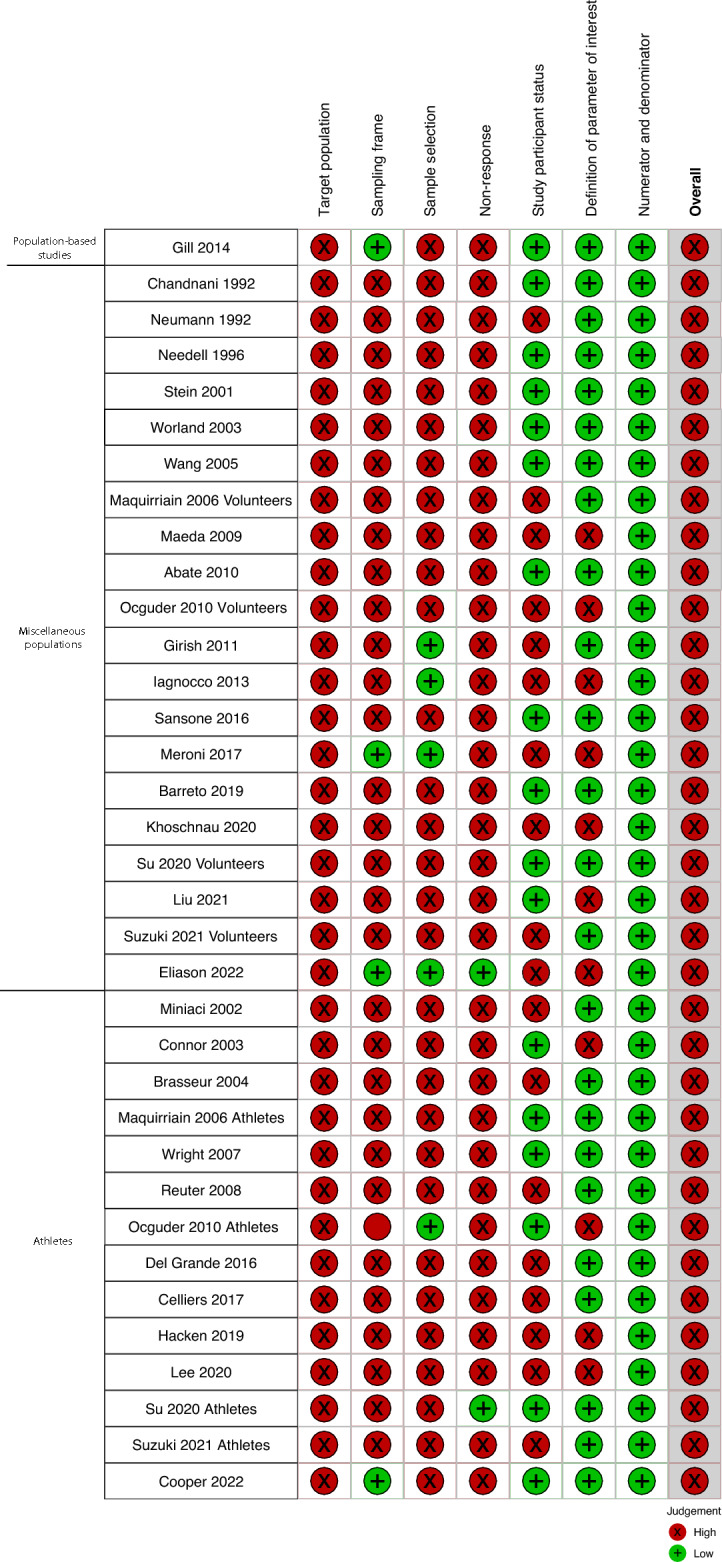
Risk of bias summary: review authors judgments about each risk of bias item for each study providing prevalence data per shoulder. All 31 studies included in the review were judged to have a high risk of bias overall

All included studies were deemed to have a high overall risk of bias, primarily due to concerns about the representativeness of the target population limiting the generalizability of the findings. This was because the populations studied were not closely representative of the national population, lacked sample frame representativeness, or did not utilize random selection or consecutive series for sample selection (Fig. 2). Additionally, there was variation in the outcome definitions across the studies (Supplementary Table 8).

### Prevalence of structural changes in asymptomatic shoulders

#### Acromioclavicular joint osteoarthritis

**Fig. 3:**
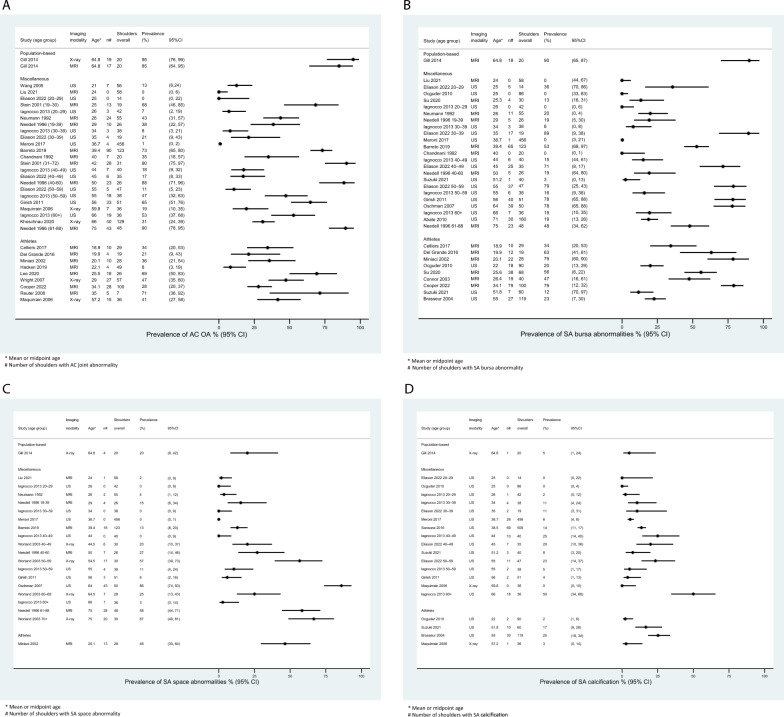
Studies reporting the prevalence of acromioclavicular osteoarthritis (AC OA) (**A**), subacromial (SA) bursa abnormalities (**B**), SA space abnormalties (**C**), and SA calcification per shoulder (**D**). Studies are arranged according to mean or midpoint age within each study population

**Fig. 4:**
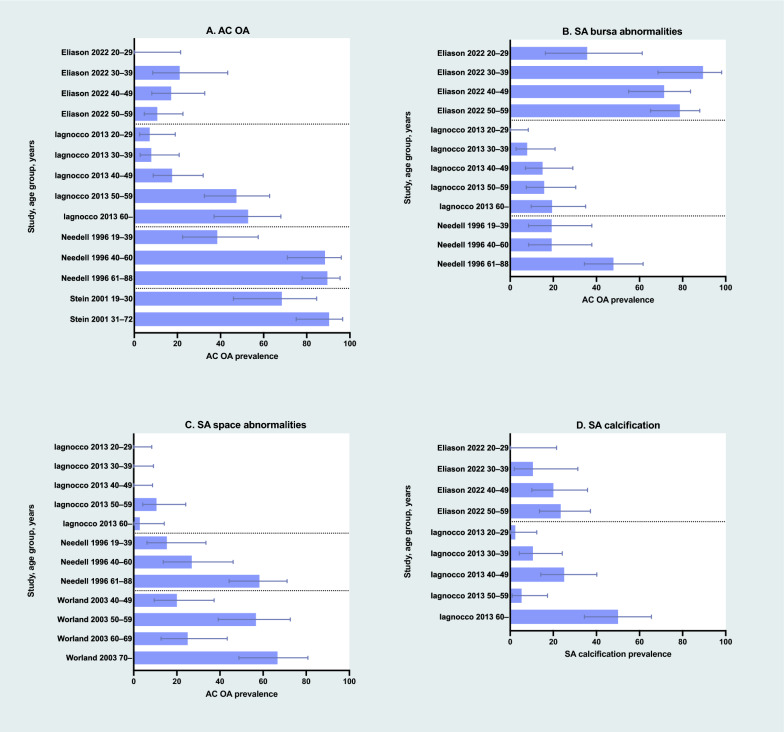
Data stratified by age group were available in four studies reporting the prevalence of acromioclavicular osteoarthritis (AC OA) (**A**), three studies on subacromial (SA) bursa abnormalities (**B**), three studies on SA space abnormalities (**C**), and two studies on SA calcification (**D**). Overall, the data suggest a trend of increasing prevalence with age

Twenty-two studies reported the prevalence of AC OA per shoulder. This included one population-based study that included both X-ray and MRI [4], 12 studies within the miscellaneous group (comprising 1 X-ray study with a mixed population of volunteers and healthcare patients [14]; 5 US studies involving healthy volunteers [6, 22], a mixed group of volunteers and athletes [16], and healthcare populations [20, 24]; 6 MRI studies with healthy volunteers [7, 26, 27, 29], volunteers with unilateral shoulder pain [5], or other musculoskeletal conditions [8]), as well as one X-ray [15] and seven MRI [32–37, 39] studies of athletes. Additionally, one X-ray study reported on both athletes and a matched cohort of volunteers [13] (Fig. 3A and Supplementary Fig. 1). Data categorized by age-group were available in four studies within the miscellaneous group [6–8, 24] (Fig. 4A).

In the population-based study (20 shoulders, mean participant age 65 years) findings consistent with AC OA were observed in 19 shoulders (95%) on X-ray and 17 shoulders (85%) on MRI [4]. Among the 21 studies with non-population-based samples (1794 shoulders; 258 X-ray, 872 US, 664 MRI; mean age 41 years), 483 shoulders (27%) had findings indicative of AC OA. The sample sizes varied from 57 to 129 shoulders in the three X-ray studies, 51 to 456 shoulders in the five US studies, and 7 to 123 shoulders in the 13 MRI studies. The prevalence of AC OA findings within the individual studies ranged from 6 to 47% for X-ray, 1 to 65% for US, and 0 to 82% for MRI.

#### Subacromial bursa abnormalities

Twenty-one studies reported the prevalence of SA bursa abnormalities per shoulder. This included one population-based study that used MRI [4], 11 studies within the miscellaneous group (comprising 6 US studies with healthy volunteers [6, 22] and healthcare populations [17, 18, 20, 24]; 5 MRI studies with healthy volunteers [7, 26, 27, 29] and volunteers with unilateral shoulder pain [5]), as well as one US [25] and five MRI [31, 33, 34, 37, 39] studies of athletes. Additionally, two US [19, 23] and one MRI [28] study reported on both athletes and a matched cohort of volunteers (Fig. 3B and Supplementary Fig. 1). Data categorized by age-group were available in three studies within the miscellaneous group [6, 7, 24] (Fig. 4B).

In the population-based study, there were MRI abnormalities in the SA bursa in 18 shoulders (90%) [4]. Among the 20 studies with non-population-based samples (2091 shoulders; 1421 US, 670 MRI; mean age 42 years), 562 (27%) shoulders had SA bursa abnormalities. Sample sizes varied from 50 to 456 in nine US studies, and 20 to 123 in 11 MRI studies and the prevalence of SA bursa abnormalities ranged from 0 to 78% for US, and 0 to 79 for MRI.

#### Subacromial space abnormalities

Eleven studies reported the prevalence of SA space abnormalities per shoulder. This included one population-based study that used X-ray [4], nine studies within the miscellaneous group (1 X-ray study with healthy volunteers [12]; 4 US studies in either healthy volunteers [6, 22] or healthcare populations [17, 20]; 4 MRI studies with either healthy volunteers [7, 27, 29] or volunteers with unilateral shoulder pain [5]), and one MRI study of athletes [39] (Fig. 3C and Supplementary Fig. 1). Data categorized by age-group were available in three studies within the miscellaneous group [6, 7, 12] (Fig. 4C).

There were X-ray SA space abnormalities in four shoulders (20%) in the population-based study [4]. Among the 10 studies with non-population-based samples (1233 shoulders; 118 X-ray, 751 US, 364 MRI; mean age 43 years), 172 shoulders (14%) shoulders showed SA space abnormalities. The sample size for the X-ray study was 118, while it ranged from 50 to 456 across four US studies and from 28 to 123 across five MRI studies. The prevalence of SA space abnormalities was 42% in the X-ray study, and ranged from 0 to 86% for US, and 2 to 46% in for MRI.

#### Subacromial calcification

Ten studies reported the prevalence of SA calcification per shoulder. This included one population-based study that used X-ray [4], five studies (all US) within the miscellaneous group comprising healthy volunteers [6, 22] and healthcare populations [20, 21, 24], and one US study of athletes [25]. Additionally, one X-ray [13] and two US studies [19, 23] reported on both athletes and a matched cohort of volunteers (Fig. 3D and RESULTS Supplementary Fig. 1). Data categorized by age-group were available in two studies within the miscellaneous group [6, 24] (Fig. 4D).

There was SA calcification in one shoulder (5%) in the population-based study [4]. Among the nine studies with non-population-based samples (1792 shoulders; 72 X-ray, 1720 US; mean age 44 years), 198 shoulders (11%) showed SA calcifications. The sample size for the X-ray study was 72, while it ranged from 51 to 509 across eight US studies. The prevalence of SA calcifications was 1% in the X-ray study and ranged from 1 to 25% in the US studies.

All prevalence estimates were judged to be of very low certainty. Detailed results of the grading process can be found in Supplementary Table 9.

### Prevalence of structural changes between asymptomatic and symptomatic shoulders

Ten studies examined the prevalence of imaging findings in both asymptomatic and symptomatic shoulders, as detailed in Supplementary Table 10. Two studies included findings from both shoulders in participants with unilateral shoulder pain [5, 24] and four studies reported on asymptomatic and symptomatic shoulders from different individuals within the same study population [4, 25, 26, 32]. The remaining studies did not clearly specify whether they reported findings within the same individuals, separate individuals, or a mix of both [14, 21, 23, 34].

#### Acromioclavicular osteoarthritis

**Fig. 5:**
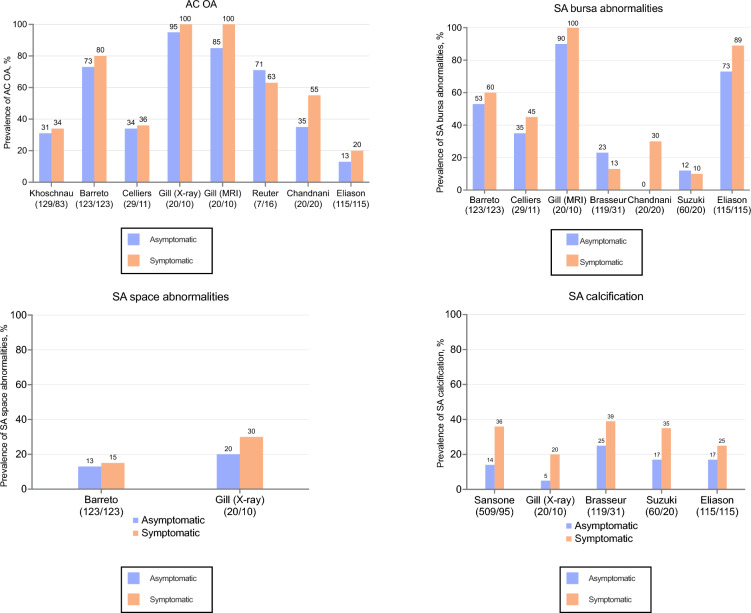
Studies reporting the prevalence of both asymptomatic and symptomatic shoulders for acromioclavicular osteoarthritis (AC OA) (**A**), subacromial (SA) bursa abnormalities (**B**), SA space abnormalities (**C**), and SA calcification per shoulder (**D**). Numbers under the authors express the total amount of shoulders (asymptomatic/symptomatic) in the study

Seven studies investigated the prevalence of AC OA, including one X-ray study [14], one US study [24], four MRI studies [5, 26, 32, 34], and one study that used both X-ray and MRI [4]. These studies collectively examined 443 asymptomatic shoulders (ranging from 7 to 129 per study) and 378 symptomatic shoulders (ranging from 10 to 123 per study). In one population-based study, the prevalence of AC OA in asymptomatic shoulders was 85% on MRI and 95% on X-ray, while in symptomatic shoulders, it was 100% on both X-ray and MRI [4]. Across all studies, the prevalence of AC OA varied from 13 to 95% in asymptomatic shoulders and from 20 to 100% in symptomatic shoulders (Fig. 5A).

#### Subacromial bursa abnormalities

Seven studies, consisting of 3 US studies [23–25] and 4 MRI studies [4, 5, 26, 34], investigated the prevalence of SA bursa abnormalities. These studies collectively examined 506 asymptomatic shoulders (sample sizes ranging from 20 to 123 per study) and 350 symptomatic shoulders (sample sizes ranging from 10 to 123 per study). In the single population-based study, the prevalence of SA bursa abnormalities was 90% in asymptomatic shoulders and 100% in symptomatic shoulders [4]. Across all studies, the prevalence of SA bursa abnormalities varied from 0 to 90% in asymptomatic shoulders and from 10 to 100% in symptomatic shoulders (Fig. 5B).

#### Subacromial space abnormalities

Two studies, one that used X-ray [4] and one that used MRI [5], investigated the prevalence of SA space abnormalities. These studies collectively examined 143 asymptomatic shoulders (ranging from 20 to 123 per study) and 133 symptomatic shoulders (ranging from 10 to 123 per study). In the single population-based study, the prevalence of SA space abnormalities was 20% in asymptomatic shoulders and 30% in symptomatic shoulders [4]. Across all studies, the prevalence of SA space abnormalities varied from 13 to 20% in asymptomatic shoulders and from 15 to 30% in symptomatic shoulders (Fig. 5C).

#### Subacromial calcification

Five studies, consisting of one X-ray [4] and four US [21, 23–25] studies, investigated the prevalence of SA calcifications. These studies collectively examined 823 asymptomatic shoulders (ranging from 20 to 509 per study) and 271 symptomatic shoulders (ranging from 10 to 115 per study). In the single population-based study, the prevalence of SA calcification was 5% in asymptomatic shoulders and 20% in symptomatic shoulders [4]. Across all studies, the prevalence of subacromial calcifications varied from 5 to 25% in asymptomatic shoulders and from 20 to 39% in symptomatic shoulders (Fig. 5D).

## Discussion

### Summary of findings

This systematic review is the first to summarize the prevalence of AC joint and SA space abnormalities in asymptomatic shoulders. We identified one population-based study and 30 additional studies with various study populations. There was considerable variation in prevalence, age groups, genders, and outcome definitions across these studies, but structural changes were frequently observed in asymptomatic shoulders in both population-based and other study populations. Overall, all studies were assessed as having a high risk of bias and their prevalence estimates were judged to be of very low certainty. The prevalence of AC joint and SA space abnormalities was nearly as high in asymptomatic shoulders as in symptomatic shoulders except for subacromial calcification, which was more prevalent in symptomatic shoulders.

### Clinical and research implications

Since imaging abnormalities are frequently observed in both asymptomatic and symptomatic shoulders, clinicians should exercise caution when linking these findings directly to a patient’s symptoms. Similar observations have been made regarding imaging findings of the glenohumeral joint [40], and in reviews of other painful musculoskeletal conditions [41–48].

Our review underscores the lack of reliable prevalence estimates for common shoulder imaging abnormalities. Our findings should therefore be interpreted with caution due to the high risk of bias of the included studies and the consequent very low certainty evidence. To establish the true age-specific prevalence of shoulder imaging abnormalities in the general population, further studies with large, representative samples are necessary. There is also a need to establish international consensus on clinically relevant outcome definitions which would facilitate better assessment of comparability across studies, and allow pooling of data across studies which would improve the precision of the prevalence estimates.

### Strengths and limitations

To our knowledge, this is the first systematic review to synthesize the prevalence of imaging abnormalities in the AC joint and SA space. Previous reviews have reported on the prevalence of abnormalities of the rotator cuff [49] and the glenohumeral joint [40], and one review has explored the link between imaging abnormalities and symptoms [50]. We conducted a comprehensive literature search covering all commonly used imaging modalities. To improve comparability, we restricted our analysis to studies comparing symptomatic and asymptomatic shoulders within the same populations. We meticulously evaluated the risk of bias for each included study using a modified version of an established risk of bias assessment tool for prevalence studies [10], and we graded the certainty of evidence for each outcome using GRADE [11].

Our review’s findings are limited by the quality of the available studies. The considerable variability in prevalence estimates across studies may be partly explained by their heterogeneity. Contributing factors include differences in study populations, potential selection bias even within the same population groups, and considerable variations in outcome definitions. Unlike findings related to the glenohumeral joint [40], age did not appear to have as large an impact on prevalence. Participants recruited from healthcare settings had a range of health conditions, such as contralateral shoulder pain [24], confirmed contralateral rotator cuff tears [17], and other healthcare issues [14, 18, 20], and the extent of upper extremity workload in athletes, may have also affected prevalence estimates.

Differences in defining symptom status may also contribute to the wide range of prevalence estimates. Some studies relied solely on symptom questionnaires or interviews, while others also included clinical examinations. Some studies included participants with prior episodes of shoulder pain while others only enrolled individuals who had never experienced shoulder symptoms. The timeframe for defining asymptomatic shoulders also varied widely; definitions ranged from “no symptoms at recruitment” to specific durations such as one week, one month, one year, or longer. Additionally, some studies did not provide a clear explanation of symptom status or timeframe.

There were also differences in how abnormalities were defined and assessed across studies. For example, AC OA definitions varied widely. Only two [8, 33] out of 14 MRI studies used the established Stein classification [8], while all included X-ray and ultrasound studies applied their own criteria which could include diverse findings such as osteophytes, joint effusion, bone oedema, joint narrowing, degeneration, hypertrophy, articular surface irregularity, sclerosis and cystic changes. Similarly, the assessment of SA bursa abnormalities differed. Criteria included bursal effusion, thickening and hypertrophy. Some studies considered size over 1 mm as abnormal [18, 33], while others considered over 2 mm as abnormal [20, 21, 23, 25, 28].

There was also variation in imaging protocols, such as differences in MRI field strength ranging from 0.25 to 3 T, which may affect the diagnostic accuracy of abnormalities.

Although we applied a method to consistently count and report abnormalities, our approach was conservative, potentially leading to underestimation of the true prevalence. Additionally, we chose to report abnormalities per shoulder rather than per person. Some studies included both shoulders from the same individual, which could have biased the prevalence estimates if they assumed that if one shoulder was structurally normal then the other would be as well. However, most studies reported prevalence per shoulder or included findings for only one shoulder per person. Therefore, we deemed it inappropriate to report prevalence per person in this review. In future studies, we recommend assessing and reporting prevalence of symptoms and imaging abnormalities on both a per shoulder and per person basis.

## Conclusion

The true prevalence of AC joint and SA space imaging abnormalities in asymptomatic individuals remains uncertain, with estimates suggesting rates as high as 90 to 95%. Except for SA calcifications, which appear more common in symptomatic shoulders, these abnormalities occur almost as frequently in asymptomatic individuals as in those with symptoms. This highlights the importance of exercising caution when attributing causation of shoulder symptoms to imaging findings.

Effective management of shoulder pain requires a comprehensive assessment of the patient’s medical history and a targeted physical examination. Imaging should be employed judiciously as a supplemental tool, primarily to confirm specific clinical suspicions or to exclude serious conditions such as tumors or infections. Finally, obtaining more accurate prevalence data is critical to guide evidence-based diagnostic and treatment strategies, ensuring appropriate interventions and minimizing unnecessary procedures.

## Supplementary Information

Supplementary file 1.

## Data Availability

No datasets were generated or analysed during the current study.

## Abbreviations

AC: Acromioclavicular
US: Ultrasound
CT: Computed tomography
MRI: Magnetic resonance imaging
OA: Osteoarthritis
SA: Subacromial
