# Supplementary material for: Imaging abnormalities of the acromioclavicular joint and subacromial space are common in asymptomatic shoulders: a systematic review
Source: J Orthop Surg Res. 2025 Jan 3;20:7. doi: 10.1186/s13018-024-05378-4 (PMC11697641; doi:10.1186/s13018-024-05378-4)
Supplement: Supplementary file 1 [file 13018_2024_5378_MOESM1_ESM.docx]

**Supplementary appendix**

**Systematic Review of Shoulder Imaging Abnormalities in Asymptomatic Adult Shoulders (SCRUTINY): Abnormalities of the Glenohumeral Joint**

**Table of contents**

[Supplementary Table 1 – Review eligibility criteria 2](#_Toc174807213)

[Supplementary Table 2 – Database searches 3](#_Toc174807214)

[Supplementary Table 3 – Risk of bias assessment 6](#_Toc174807215)

[Supplementary Table 4 – GRADE tool used in this study 11](#_Toc174807216)

[Supplementary Table 5 – Deviations from study methods 12](#_Toc174807217)

[Supplementary Table 6 – Reports excluded from the review 13](#_Toc174807218)

[Supplementary Table 7 – Included studies with unusable prevalence data 17](#_Toc174807219)

[Supplementary Table 8 – Details on study population, participant recruitment, and outcomes 19](#_Toc174807220)

[Supplementary Table 9 – The certainty of evidence 27](#_Toc174807221)

[Supplementary Table 10 – Comparison of asymptomatic and symptomatic shoulders 28](#_Toc174807222)

[Supplementary Figure 1 – Scatter plots of athletes vs. non-athletes 30](#_Toc174807223)

# Supplementary Table 1 – Review eligibility criteria

| We will include studies of any design reporting the prevalence of abnormalities (however defined) in adults whose shoulder**s** were asymptomatic (however defined) at the time of imaging (with or without previous episode/s of shoulder injury or symptoms). Eligible studies may;  -purposefully recruit adults whose shoulder or shoulders are asymptomatic  -determine shoulder symptom status during the course of the study (it is not known at recruitment) and report prevalence in shoulders that are found to be asymptomatic (however defined) and symptomatic (however defined) at the time of imaging  When studies perform imaging of the shoulder and incorporate a method of determining shoulder symptom status, but do not report prevalence data by shoulder status (symptomatic or asymptomatic), the study authors will be contacted for data. If data is provided, the study will be included in the review.  Additional inclusion criteria are studies that;   1. primarily evaluate adults (mean age of the study population ≥18 years or data for a subset of the population ≥18 years is provided) 2. are published in any language 3. are only available in full text 4. image shoulders with either MRI, US, x-ray or CT 5. image >1 asymptomatic shoulder |
| --- |
| We will exclude studies that;   1. report prevalence only in shoulders that are symptomatic 2. evaluate children and adolescents only or mean age of the study population ≤18 years 3. are not available in full text (e.g. conference abstracts, poster presentations) 4. report only morphological measures of shoulder structure outcomes |

#

# Supplementary Table 2 – Database searches

| Searches of bibliographic databases including Ovid MEDLINE, Embase, CINAHL and Web of Science were conducted on 12/06/2023. The database provider, coverage and search strings used for each database are provided below. No limitations or search filters were applied. |
| --- |
| **MEDLINE (via OVID, 1950 to 12/06/2023)**   \| 1 \| exp SHOULDER/ \| \| --- \| --- \| \| 2 \| shoulder*.ab,ti. \| \| 3 \| exp Rotator Cuff/ \| \| 4 \| rotator.ab,ti. \| \| 5 \| 1 or 2 or 3 or 4 \| \| 6 \| Magnetic Resonance Imaging/ \| \| 7 \| "MR imag*".ti,ab. \| \| 8 \| "magnetic resonance imag*".ab,ti. \| \| 9 \| MRI.ab,ti. \| \| 10 \| ULTRASONOGRAPHY/ \| \| 11 \| ultrasound.ab,ti. \| \| 12 \| ultrasonograph*.ab,ti. \| \| 13 \| sonograph*.ab,ti. \| \| 14 \| US.ab,ti. \| \| 15 \| X-Rays/ \| \| 16 \| "x-ray*".ab,ti. \| \| 17 \| xray*.ab,ti. \| \| 18 \| "x ray*".ab,ti. \| \| 19 \| radiograph*.ab,ti. \| \| 20 \| Tomography, X-Ray Computed/ \| \| 21 \| "comput* tomograph*".ab,ti. \| \| 22 \| "computer aided tomograph*".ab,ti. \| \| 23 \| "computer assisted tomograph*".ab,ti. \| \| 24 \| CT.ab,ti. \| \| 25 \| "CAT scan*".ab,ti. \| \| 26 \| PREVALENCE/ \| \| 27 \| prevalen*.ab,ti. \| \| 28 \| inciden*.ab,ti. \| \| 29 \| frequency.ab,ti. \| \| 30 \| ((findings or abnormal*) adj5 (image or images or imaging or shoulder* or rotator)).ti,ab. \| \| 31 \| 6 or 7 or 8 or 9 or 10 or 11 or 12 or 13 or 14 or 15 or 16 or 17 or 18 or 19 or 20 or 21 or 22 or 23 or 24 or 25 \| \| 32 \| 26 or 27 or 28 or 29 or 30 \| \| 33 \| asymptomatic.ab,ti. \| \| 34 \| symptomatic.ab,ti. \| \| 35 \| symptoms.ab,ti. \| \| 36 \| risk.ti. \| \| 37 \| uninjured.ab,ti. \| \| 38 \| "pain free".ab,ti. \| \| 39 \| "healthy volunteers".ab,ti. \| \| 40 \| 33 or 34 or 35 or 36 or 37 or 38 or 39 \| \| 41 \| 5 and 31 and 32 and 40 \| \| 42 \| exp SHOULDER/ \| \| 43 \| shoulder*.ab,ti. \| \| 44 \| exp Rotator Cuff/ \| \| 45 \| rotator.ab,ti. \| \| 46 \| 42 or 43 or 44 or 45 \| \| 47 \| Magnetic Resonance Imaging/ \| \| 48 \| "MR imag*".ti,ab. \| \| 49 \| "magnetic resonance imag*".ab,ti. \| \| 50 \| MRI.ab,ti. \| \| 51 \| ULTRASONOGRAPHY/ \| \| 52 \| ultrasound.ab,ti. \| \| 53 \| ultrasonograph*.ab,ti. \| \| 54 \| sonograph*.ab,ti. \| \| 55 \| US.ab,ti. \| \| 56 \| X-Rays/ \| \| 57 \| "x-ray*".ab,ti. \| \| 58 \| xray*.ab,ti. \| \| 59 \| "x ray*".ab,ti. \| \| 60 \| radiograph*.ab,ti. \| \| 61 \| Tomography, X-Ray Computed/ \| \| 62 \| "comput* tomograph*".ab,ti. \| \| 63 \| "computer aided tomograph*".ab,ti. \| \| 64 \| "computer assisted tomograph*".ab,ti. \| \| 65 \| CT.ab,ti. \| \| 66 \| "CAT scan*".ab,ti. \| \| 67 \| PREVALENCE/ \| \| 68 \| prevalen*.ab,ti. \| \| 69 \| inciden*.ab,ti. \| \| 70 \| frequency.ab,ti. \| \| 71 \| ((findings or abnormal*) adj5 (image or images or imaging or shoulder* or rotator)).ti,ab. \| \| 72 \| 47 or 48 or 49 or 50 or 51 or 52 or 53 or 54 or 55 or 56 or 57 or 58 or 59 or 60 or 61 or 62 or 63 or 64 or 65 or 66 \| \| 73 \| 67 or 68 or 69 or 70 or 71 \| \| 74 \| asymptomatic.ab,ti. \| \| 75 \| symptomatic.ab,ti. \| \| 76 \| symptoms.ab,ti. \| \| 77 \| risk.ti. \| \| 78 \| uninjured.ab,ti. \| \| 79 \| "pain free".ab,ti. \| \| 80 \| "healthy volunteers".ab,ti. \| \| 81 \| 74 or 75 or 76 or 77 or 78 or 79 or 80 \| \| 82 \| 46 and 72 and 73 and 81 \| |
| **Embase (via Elsevier, 1974 to 12/06/2023)**  ('SHOULDER'/exp OR shoulder*:ti,ab OR 'Rotator Cuff'/exp OR rotator:ti,ab)  AND  ((asymptomatic OR symptomatic OR symptoms):ti,ab OR risk:ti)  AND  ('nuclear magnetic resonance imaging'/de OR "magnetic resonance imag*":ti,ab OR MRI:ti,ab OR 'echography'/de OR ultrasound:ti,ab OR ultrasonograph*:ti,ab OR sonograp*:ti,ab OR US:ti,ab OR 'X ray'/de OR "x ray*":ti,ab OR x-ray*:ti,ab OR xray*:ti,ab OR radiograph*:ti,ab OR 'x-ray computed tomography'/de OR "comput* tomograph*":ti,ab OR "computer aided tomograph*":ti,ab OR "computer assisted tomograph*":ti,ab OR CT:ti,ab OR "CAT scan*":ti,ab)  AND  ('PREVALENCE'/de OR inciden*:ti,ab OR frequency:ti,ab OR ((findings OR abnormal*) NEAR/5 (image* OR imaging OR shoulder* OR rotator)):ti,ab) |
| **CINAHL (Cumulative Index to Nursing and Allied Health Literature) (via EBSCO, 1981 to 12/06/2023)**  ((MH "SHOULDER+") OR TI shoulder* OR AB shoulder* OR (MH "Rotator Cuff+") OR TI rotator OR AB rotator)  AND  ((TI asymptomatic OR AB asymptomatic OR TI symptomatic OR AB symptomatic OR TI symptoms OR AB symptoms) OR TI risk)  AND  ((MH "Magnetic Resonance Imaging") OR TI "magnetic resonance imag*" OR AB "magnetic resonance imag*" OR TI MRI OR AB MRI OR (MH "ULTRASONOGRAPHY") OR TI ultrasound OR AB ultrasound OR TI ultrasonograph* OR AB ultrasonograph* OR TI sonograp* OR AB sonograp* OR TI US OR AB US OR (MH "X-Rays") OR TI "x ray*" OR AB "x ray*" OR TI x-ray* OR AB x-ray* OR TI xray* OR AB xray* OR TI radiograph* OR AB radiograph* OR (MH "Tomography, X-Ray Computed") OR TI "comput* tomograph*" OR AB "comput* tomograph*" OR TI "computer aided tomograph*" OR AB "computer aided tomograph*" OR TI "computer assisted tomograph*" OR AB "computer assisted tomograph*" OR TI CT* OR AB CT* OR TI "CAT scan*" OR AB "CAT scan*")  AND  ((MH "PREVALENCE") OR TI inciden* OR AB inciden* OR TI frequency OR AB frequency OR ((TI findings OR AB findings OR TI abnormal* OR AB abnormal*) N5 (TI image* OR AB image* OR TI imaging OR AB imaging OR TI shoulder* OR AB shoulder* OR TI rotator OR AB rotator))) |
| **Web of Science Core Collection (via Clarivate Analytics, 1900 to 12/06/2023)**  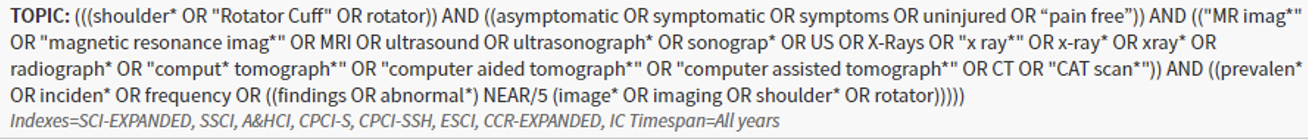 |

# Supplementary Table 3 – Risk of bias assessment

For the purposes of this review, we adapted the tool of Hoy D, Brooks P, Woolf A, Blyth F, March L, Bain C, et al. Assessing risk of bias in prevalence studies: modification of an existing tool and evidence of interrater agreement. J Clin Epidemiol. 2012;65: 934-939.

Adaptions include;

- Rewording of item 3 to include consecutive series as well as random selection as acceptable means of sample selection.
- Removal of item 5 from the original tool (Were data collected directly from the subjects?) As data were collected directly from participants in all studies, the item was considered unnecessary and was removed
- Rewording of item 6 in the original tool ‘Was an acceptable case definition used in the study?’ to ‘Was an acceptable definition of study participant status used in the study?’
- Removal of item 7 (Was the study instrument that measured the parameter of interest shown to have reliability and validity?) from the original tool. We decided to assume that the imaging modalities evaluated in this review are reliable and valid.
- Removal of item 8 (Was the same mode of data collection used for all subjects?) Variation in the procedure for performing US is likely, but less so for MRI. Consequently, we decided to remove this item from the risk of bias tool but will record and present details of the US method (procedure for performing the US, number of operators, experience and blinding of operator are currently in the data extraction form)
- Removal of item 9 ‘Was the length of the shortest prevalence period for the parameter of interest appropriate?’

| **Original tool criterion** | **Adapted tool** | | |
| --- | --- | --- | --- |
| **Risk of bias item** | **Risk of bias item** | **Risk of bias levels** | **Interpretation/Application for this review** |
| External validity | | | |
| 1. Was the study’s target population a **close representation** of the national population in relation to relevant variables, e.g. age, sex, occupation | 1. Was the study’s target population a **close representation** of the national population in relation to relevant variables, e.g. age, sex, occupation | **Yes (LOW RISK)**: The study’s target population was a close representation of the national population | Judge this criteria according to whether the study population is a close representation of all people with asymptomatic shoulders i.e. the general population with a range of age and genders. Studies in specific populations such as athletes or people attending for treatment at an orthopaedic clinic for example would be rated at high risk for this criterion. |
|  |  | **NO (HIGH RISK)**: The study’s target population was clearly NOT representative of the national population |  |
|  |  | **NO (HIGH RISK – INSUFFICIENT INFORMATION)**: There is insufficient information to permit a judgment about this item |  |
| 2. Was the sampling frame a **true or close representation** of the target population? | 2. Was the sampling frame a **true or close representation** of the target population? | **YES (LOW RISK)**: The sampling frame was a true or close representation of the target population | Judge this criterion according to whether the study sampling frame is likely to be reflective of the target population. E.g. if the target population is older men, is the group sampled likely to be reflective of all older men or is it only a select subgroup from a healthcare population? If for example the study target population is people with orthopaedic conditions, and the sampling frame includes participants from >1 centres/hospitals and these participants are representative of the target population in terms of age (e.g. wide range of ages) and gender (even gender split) the study may be rated as low risk on this criterion. If the study includes participants from only 1 centre/hospital/medical unit the study should be rated as high risk on this criterion. |
|  |  | **NO (HIGH RISK)**: The sampling frame was not a true or close representation of the target population |  |
|  |  | **NO (HIGH RISK – INSUFFICIENT INFORMATION)**: There is insufficient information to permit a judgment about this item |  |
|  |  | Explanation from the original tool: The sampling frame is a list of the sampling units in the target population and the study sample is drawn from this list. Examples: The sampling frame was a list of almost every individual within the target population = Yes (Low risk); The cluster sampling method was used and the sample of clusters/villages was drawn from a list of all villages in the target population = Yes (Low risk); The sampling frame was a list of just one particular ethnic group within the overall target population, which comprised many groups = No (High risk) |  |
| 3. Was some form of **random selection** used to select the sample, OR, was a census undertaken? | 3. Was some form of **random selection or consecutive series** used to select the sample, OR, was a census undertaken? | **YES (LOW RISK)**: A census was undertaken, OR, some form of random selection or consecutive series was used to select the sample | If the study uses a method of random selection to recruit participants or uses a consecutive series or census approach this criterion would be judged as low risk.  If the study reports using a convenience sample (e.g. volunteers) this criterion would be judged as high risk. |
|  |  | **NO (HIGH RISK)**: A census was NOT undertaken, AND some form of random selection or consecutive series was NOT used to select the sample |  |
|  |  | **NO (HIGH RISK – INSUFFICIENT INFORMATION)**: There is insufficient information to permit a judgment about this item |  |
|  |  | Explanation from the original tool: A census collects information from every unit in the sampling frame. In a survey, only part of the sampling frame is sampled. In these instances, random selection of the sample helps minimise study bias. Examples: The sample was selected using simple random sampling = Yes (Low risk); The target population was the village and every person in the village was sampled = Yes (Low risk); The sampling frame was a list of just one particular ethnic group within the overall target poulation, which comprised many groups = No (High risk) |  |
| 4. Was the likelihood of non-response bias minimal? | 4. Was the likelihood of non-response bias minimal? | **YES (LOW RISK)**: The response rate for the study was >/=75% OR, an anlysis was performed that showed no significant difference in relevant demographic characteristics between responders and non-responders | There may be concern around non-response bias if it is not reported how many potential participants were identified as eligible or invited to participate in the study.  If a study reports only the number of people included in the study and not the number eligible or invited, it is rated as high risk (insufficient information).  If a study reports both the number of people eligible or invited to participate, and the number who then did participate, and this proportion is >/= 75% ,the study may be rated as low risk.  If a study reports both the number of people eligible or invited to participate, and the number who then did participate, and this proportion is <75%, the study may be rated as high risk.  If the study reports including a ‘consecutive series’ or a ‘random selection’, the number within that series/selection who did not participate should also be reported (or it should be clearly reported that all in the series or selection participated in which case it would be judged as low risk)  If a study using random selection or consecutive sample reports only the number of people included in the study and not the number selected or in the series, it is rated as high risk (insufficient information).  If a study using random selection or consecutive sample reports both the number selected or in the series and the number that were included in the study and this proportion is >/= 75%, the study is rated as low risk.  If a study using random selection or consecutive sample reports both the number selected or in the series and the number that were included in the study and this proportion was <75%, the study is rated as high risk on this criterion. |
|  |  | **NO (HIGH RISK)**: The response rate was <75% and if any analysis comparing responders and non-responders was done, it showed a significant difference in relevant characteristics between responders and non-responders. |  |
| Internal validity | | | |
| 5. Was data collected **directly from the subjects** (as opposed to a proxy)? | 5. Was an **acceptable definition of study participant status** used in the study? | **Yes (LOW RISK)**: An acceptable definition of study participant status was used | This criterion relates to the asymptomatic or symptomatic status of study participants. For studies addressing Review Question 2 (prevalence comparison between symptomatic and asymptomatic populations) this criterion will be answered for each population  An appropriate definition of an asymptomatic population would include no past history of any shoulder condition, trauma or treatment including surgery and no current symptoms (rate as Low risk).  If there is insufficient information to judge, rate as high risk (insufficient information) |
|  |  | **NO (HIGH RISK)**: An acceptable definition of study participant status was not used |  |
|  |  | **NO (HIGH RISK – INSUFFICIENT INFORMATION)**: There is insufficient information to permit a judgment about this item |  |
| 6. Was an acceptable case definition used in the study? | 6. Was an **acceptable definition of the parameter of interest** (the abnormality X or Y) used in the study? | **YES (LOW RISK)**: An acceptable definition of the paratmeter of interest was used | This criterion relates to the definition used to determine the presence of an abnormality.  If the study provides an adequate description of the definition/criteria used to determine the presence of an abnormality and this definition would be generally acceptable to clinicians, this criterion may be rated low risk.  If the definition/criterion used to define the abnormality would not be considered acceptable the study may be rated as high risk.  If no definition/criterion is provided for how an abnormality is defined the study may be rated as high risk (insufficient information) on this criterion.  When studies are reporting multiple abnormalities, the acceptability of the definitions could be judged overall or judgment should be based on the definition provided for the abnormality of greatest clinical relevance |
|  |  | **NO (HIGH RISK)**: An acceptable definition of the parameter of interest was not used |  |
|  |  | **NO (HIGH RISK – INSUFFICIENT INFORMATION)**: There is insufficient information to permit a judgment about this item |  |
| 7. Was the study instrument that measured the parameter of interest (e.g. prevalence of low back pain) shown to have **reliability and validity** (if necessary)? | Not included for this review |  |  |
| 8. Was the **same mode of data collection** used for all subjects? | Not included for this review |  |  |
| 9. Was the **length of the shortest prevalence period** for the parameter of interest appropriate? | Not included for this review |  |  |
| 10. Were the **numerator(s) and denominator(s)** for the parameter of interest appropriate? | 7.Were the **numerator and denominator(s)** for the parameter of interest appropriate? | **YES (LOW RISK)**: The paper presented appropriate numerator(s) AND denominator(s) for the parameter of interest | Studies reporting prevalence data by participant rather than shoulder are rated as high risk of bias and will be presented separately. We will attempt to contact authors to obtain data per shoulder. |
|  |  | **NO (HIGH RISK)**: The paper did present numerator(s) AND denominator(s) for the parameter of interest but one of more of these were inappropriate |  |
|  |  | **NO (HIGH RISK)**: There is insufficient information to permit a judgment about this item |  |
|  | Overall risk of study bias | | |
|  | **LOW RISK OF BIAS**: Further research is very unlikely to change our confidence in the estimate  **MODERATE RISK OF BIAS**: Further research is likely to have an important impact on our confidence in the estimate and may change the estimate  **HIGH RISK OF BIAS**: Further research is very likely to have an important impact on our confidence in the estimate and is likely to change the estimate | | Judgment here is based primarily on risk of bias in key criteria.  We are primarily interested in determining the prevalence of abnormalities in adults of a range of ages and gender with no shoulder pain at the time of imaging (with no history of pain, treatment, or trauma) residing within a country or a region that closely represents the national population.  Therefore, the key criterion for this review is;  - The studies target population is a close representation of the national population (Criterion 1) If the study is not considered to attempt to recruit a population that closely represents the national population, the study cannot be rated as low risk overall. Study populations that would be rated as high risk include those constituting specific types of people e.g. elite athletes or people of a particular occupation or recruiting people in a particular setting such as a healthcare setting. Studies that may be rated low risk on this criterion include those whose target population includes people residing in a defined geographical area (population based) or that are recruited using community based recruitment strategies such as advertisements in local newspapers or studies who attempt to recruit a mix of adults from various settings. The studies considered to include a nationally representative population may be considered to be moderate or low risk overall if the study sampling frame also represents closely the target/national population and a random or consecutive sample is used. For these studies a decision on whether the overall rating of risk of bias is moderate or low will depend on consideration of other criteria such as study selection and non-response and the decision will be achieved through discussion. Studies whose target population closely represents the national population but whose sampling frame is not representative of that population cannot be rated low risk of bias. |

# Supplementary Table 4 – GRADE tool used in this study

|  | **Assessment of the domain** | |
| --- | --- | --- |
| **Domain of GRADE** | **Original tool for prognostic studies (Iorio et al. BMJ 2015)** | **Adapted tool in this prevalence study** |
| Risk of bias | Primary  • Was there a representative and well-defined sample of patients:  – Who did not have the outcome of interest at the time of initial observation?  – Who were at a similar, identifiable, common, and possible early point in the  course of the disease?  • Was follow-up sufficiently long and complete?  Secondary  • Were objective and unbiased outcome criteria used?  • Were all characteristics of patients known or suspected to affect the outcome  recorded?  • Was there adjustment for important prognostic factors? | The **overall risk of bias** of the studies at each category (i.e., study population and imaging modality) was assessed. If risk of bias was deemed high, the certainty of evidence was downgraded 1 or 2 levels. |
| Inconsistency | Variability in point estimates, extent of overlap in confidence intervals, and where point estimates lie in relation to decision thresholds. | If there was **unexplained** **variability** in the point estimates between the studies or 95% CIs were not overlapping, we downgraded the certainty of evidence 1 or 2 levels. In case there was only a one study in the category, the certainty of evidence was not downgraded. |
| Imprecision | The width of the 95% confidence interval around the pooled estimate and the position of the confidence interval relative to a clinical decision threshold. The GRADE rule for prognosis is to rate down confidence in estimates of the event rate if the effect on the patient, or clinical action, would differ depending on whether the upper or the lower boundary of the confidence interval represented the truth. | We made the judgement based on the **overall number of shoulders** in each category. We downgraded the certainty of evidence by 1 in categories with under 500 shoulders and by 2 in categories with under 50 shoulders. |
| Indirectness | Does the studied population correspond to the population of interest and does the measured outcome capture what is believed important? | If the population sample in the category was **not representative for the target population**, we downgraded the certainty of evidence by 1. |
| Publication bias | Statistical tests (Egger’s test when when heterogeneity is low and data are normally distributed **or** Begg’s test when studies have an asymmetric distribution and inconsistency of results is high) for publication bias. | We made a judgement that there are **no obvious reasons for publication bias in prevalence studies** and thus did not downgrade any of the categories for this dimension. |

# Supplementary Table 5 – Deviations from study methods

Deviations from study methods specified at review protocol registration

| **Method specified** | **Deviation, with justification** |
| --- | --- |
| The protocol specified that if possible, prevalence estimates from each imaging modality would be pooled across studies and meta-regression conducted to estimate the effects of age and sex on prevalence estimates. | Large heterogeneity was observed in the study populations and prevalence estimates of the included studies. As such pooling of prevalence estimates was not considered appropriate. Meta-regression was conducted using explanatory variables specified prior to the analysis. The entire body of studies were summarised descriptively. |
| The abnormalities to be reported in the review were not pre-specified in the protocol. | Due to the large number of abnormalities reported in the eligible studies it was necessary to limit reporting of abnormalities to those considered of greatest clinical relevance. This was achieved through discussion between clinician reviewers. |
| The prevalence of abnormalities in asymptomatic shoulders with and without a history of symptoms was to be reported. | As few studies provided data according to the previous symptoms this information was not presented in the review. In the few studies providing this data, data from the asymptomatic no history and asymptomatic with history study groups were combined. |
| A PubMed related citations search of the included studies was to be conducted | Due to the large number of studies included in the review and resources limitations, this method of searching was not conducted. |

# Supplementary Table 6 – Reports excluded from the review

Reports excluded from the review with reason for exclusion (n=93)

| 1. | Abdelzaher MG, Tharwat S, Abdelkhalek A, Abdelsalam A. Role of ultrasound in detection of shoulder joint pathologies in asymptomatic rheumatoid arthritis patients. Annals of the Rheumatic Diseases. 2020; 79(SUPPL 1):1825. | Not population of interest |
| --- | --- | --- |
| 2. | Akbar M, Brunner M, Balean G, Grieser T, Bruckner T, Loew M, et al. Etiology of rotator cuff tears in paraplegic patients: a case-control study.[Retraction in J Shoulder Elbow Surg. 2012 Jul;21(7):983; PMID: 22608702]. J Shoulder Elbow Surg. 2012;21(1):23-8. | Not a study of the prevalence of imaging abnormalities |
| 3. | Akbar M, Brunner M, Balean G, Grieser T, Bruckner T, Loew M, et al. RETRACTED: Etiology of rotator cuff tears in paraplegic patients: a case-control study (Retracted article. See vol. 21, pg. 983, 2012). Journal of Shoulder and Elbow Surgery. 2012;21(1):23-8. | Not a study of the prevalence of imaging abnormalities |
| 4. | Akbar M, Brunner M, Balean G, Grieser T, Bruckner T, Loew M, et al. Etiology of rotator cuff tears in paraplegic patients: A case-control study. Journal of Shoulder and Elbow Surgery. 2012;21(1):23-8. | Duplicate, poster or conference abstract only |
| 5. | Alasaarela EM, Alasaarela ELI. Ultrasound evaluation of painful rheumatoid shoulders. J Rheumatol. 1994;21(9):1642-8. | Not population of interest |
| 6. | Andreotti C, Ghobert AD, Della Sala SW, Morini A. Shoulder pain in hemodialysis patients: Multidisciplinary approach to diagnosis and treatment. Giornale Italiano di Nefrologia. 1997;14(4):229-37. | Not population of interest |
| 7. | Arvikar SL, Lin J, Kohler MJ. Musculoskeletal ultrasound reveals calcific deposition arthropathy in seronegative inflammatory arthritis patients. Arthritis and Rheumatology. 2014;66:S77. | Duplicate, poster or conference abstract only |
| 8. | Atala NA, Bongiovanni SL, Galich AM, Bruchmann MG, Rossi LA, Tanoira I, Ranalletta M. Is sarcopenia a risk factor for rotator cuff tears? Journal of Shoulder & Elbow Surgery. 2021 Aug; 30(8):1851-1855. | No outcomes of interest |
| 9. | Balke M, Schmidt C, Dedy N, Banerjee M, Bouillon B, Liem D. Correlation of acromial morphology with impingement syndrome and rotator cuff tears. Acta Orthop. 2013;84(2):178-83. | No outcomes of interest |
| 10. | Beitzel K, Zandt J, Beitzel KI, Buchmann S, Hahn D, Schwirtz A, et al. Clinical and 3T MRI evaluation of high performance junior elite overhead athletes: Basis for a preventive shoulder training. Arthroscopy - Journal of Arthroscopic and Related Surgery. 2011;27(10):e202. | Not population of interest |
| 11. | Beitzel K, Zandt JF, Buchmann S, Beitzel KI, Schwirtz A, Imhoff AB, et al. Structural and biomechanical changes in shoulders of junior javelin throwers: a comprehensive evaluation as a proof of concept for a preventive exercise protocol. Knee Surgery Sports Traumatology Arthroscopy. 2016;24(6):1931-42. | Duplicate, poster or conference abstract only |
| 12. | Bellam K, Taori K, Disawal A. High-resolution ultrasound vs MRI for cuff and non-cuff shoulder pathologies. Journal of Medical Imaging and Radiation Oncology. 2012;56:56. | Not population of interest |
| 13. | Blauwet CA, Chakraverty J, Derman W, Idrisova G, Martin P, Miller SC, et al. Shoulder Pain, Function, and Ultrasound-Determined Structure in Elite Wheelchair-Using Para Athletes: An Observational Study. Medicine & Science in Sports & Exercise. 2022; 54(6):896-904. | No outcomes of interest |
| 14. | Boersma E, Crijns T, Nijhuis-van der Sanden M, Edwards M, Ring D, Janssen S. Accuracy and reliability of MRI-reports to determine which shoulder is symptomatic for workers compensation patients with unilateral symptoms. Journal of Orthopaedics. 2020 Sep-Oct; 21:199-202. | No outcomes of interest |
| 15. | Bourji K, Mecoli C, Paik J, Albayda J, Tiniakou E, Kelly W, et al. Avascular Necrosis in the Hopkins Myositis Cohort: A Single Center Experience. Arthritis and Rheumatology. 2020; 72(SUPPL 10):2193-2195. | No outcomes of interest |
| 16. | Bourji KI, Mecoli CA, Paik JJ, Albayda J, Tiniakou E, Kelly W, et al. Prevalence of avascular necrosis in idiopathic inflammatory myopathies: a single-centre experience. Rheumatology. 2022; 61(3):936-942. | No outcomes of interest |
| 17. | Brogan DM, Carofino BC, Kircher MF, Spinner RJ, Elhassan BT, Bishop AT, et al. Prevalence of Rotator Cuff Tears in Adults with Traumatic Brachial Plexus Injuries. Journal of Bone and Joint Surgery-American Volume. 2014;96A(16). | Not population of interest |
| 18. | Burg LC, Karakostas P, Behning C, Brossart P, Kermani TA, Schafer VS. Prevalence and characteristics of giant cell arteritis in patients with newly diagnosed polymyalgia rheumatica - a prospective cohort study. Therapeutic Advances in Musculoskeletal Disease. 2023; 15:1759720X221149963. | No outcomes of interest |
| 19. | Burk Jr DL, Torres JL, Marone PJ, Mitchell DG, Rifkin MD, Karasick D. MR imaging of shoulder injuries in professional baseball players. Journal of magnetic resonance imaging : JMRI. 1991;1(3):385-9. | Not population of interest |
| 20. | Canon A, Roy L, Chevalier X, Giraudier S, Eymard F. Calcific tendinopathy: an unexpected side effect of tyrosine kinase inhibitor? Leukemia & Lymphoma. 2022 12; 63(13):3175-3180. | Not population of interest |
| 21. | Cartland JP, Crues JV, 3rd, Stauffer A, Nottage W, Ryu RK. MR imaging in the evaluation of SLAP injuries of the shoulder: findings in 10 patients. AJR Am J Roentgenol. 1992;159(4):787-92. | Not population of interest |
| 22. | Chávez-López M, Alvarez Del Castillo-Araujo AL, Monroy-Guizar E, Valerio M, López-López F. Shoulder ultrasound in women with breast cancer and mastectomy. Annals of the Rheumatic Diseases. 2013;72. | Duplicate, poster or conference abstract only |
| 23. | Cho HJ, Morey V, Kang JY, Kim KW, Kim TK. Prevalence and Risk Factors of Spine, Shoulder, Hand, Hip, and Knee Osteoarthritis in Community-dwelling Koreans Older Than Age 65 Years. Clinical Orthopaedics & Related Research. 2015;473(10):3307-14. | Not population of interest |
| 24. | Clavert P, Sirveaux F, Soc Francaise A. Shoulder calcifying tendinitis. Revue De Chirurgie Orthopedique Et Reparatrice De L Appareil Moteur. 2008;94(8):S336-S55. | Not population of interest |
| 25. | Coates PTH, Slavotinek JP, McDonald SP, Disney APS. MRI findings in shoulders of symptomatic chronic hemodialysis patients. Kidney International. 1997;51(4):1316-7. | Duplicate, poster or conference abstract only |
| 26. | Dai SM, Han XH, Zhao DB, Shi YQ, Liu Y, Meng JM. Prevalence of rheumatic symptoms, rheumatoid arthritis, ankylosing spondylitis, and gout in Shanghai, China: a COPCORD study. J Rheumatol. 2003;30(10):2245-51. | Not a study of the prevalence of imaging abnormalities |
| 27. | de Witte PB, van der Zwaal P, Visch W, Schut J, Nagels J, Nelissen RG, et al. Arm adductor with arm abduction in rotator cuff tear patients vs. healthy -- design of a new measuring instrument [corrected].[Erratum appears in Hum Mov Sci. 2013 Aug;32(4):875-6]. Hum Mov Sci. 2012;31(2):461-71. | Not a study of the prevalence of imaging abnormalities |
| 28. | Ekeberg OM, Bautz-Holter E, Juel NG, Engebretsen K, Kvalheim S, Brox JI. Clinical, socio-demographic and radiological predictors of short-term outcome in rotator cuff disease. Bmc Musculoskeletal Disorders. 2010;11. | Not population of interest |
| 29. | Farley TE, Neumann CH, Steinbach LS, Petersen SA. The coracoacromial arch: MR evaluation and correlation with rotator cuff pathology. Skeletal Radiol. 1994 Nov;23(8):641-5. | Duplicate, poster or conference abstract only |
| 30. | Finley M, Euiler E, Trojian T, Gracely E, Schmidt-Read M, Frye SK, et al. Shoulder impairment and pain of individuals with newly acquired spinal cord injury compared to uninjured peers. Spinal Cord Series and Cases. 2020 08 04; 6(1):68. | No outcomes of interest |
| 31. | Girish G, Lobo LD, Boon T, Robertson B, Morag Y, Fessell D, et al. Abnormal Sonographic Findings of Asymptomatic Shoulder Scans. American Journal of Roentgenology. 2010;194(5). | Duplicate, poster or conference abstract only |
| 32. | Gokalp G, Algin O, Yildirim N, Yazici Z. Adhesive capsulitis: contrast-enhanced shoulder MRI findings. J Med Imaging Radiat Oncol. 2011;55(2):119-25. | Not population of interest |
| 33. | Goldhaber NH, Lee C, Davis S, Wosmek J, Brock A, Stetson W. MRI findings in asymptomatic elite overhead athletes. Journal of Investigative Medicine. 2015;63(1):193-4. | Duplicate, poster or conference abstract only |
| 34. | Goodman RS. Abnormal findings on magnetic resonance images of asymptomatic shoulders. J Bone Joint Surg Am. 1996;78(4):633. | Not a study of the prevalence of imaging abnormalities |
| 35. | Hanaoka M, Miyanaga Y, Shiraki H, Mukai N, Miyakawa S. The relationships between shoulder's function and damage for handball players. Japanese Journal of Physical Fitness and Sports Medicine. 2005;54(2):179-84. | No outcomes of interest |
| 36. | Hayashi D, Gould E, Shroyer R, van Staalduinen E, Yang J, Mufti M, Huang M. Shoulder adhesive capsulitis in cancer patients undergoing positron emission tomography - computed tomography and the association with shoulder pain. World Journal of Radiology. 2021 Oct 28; 13(10):344-353. | No outcomes of interest |
| 37. | Hirata A, Ogura T, Hayashi N, Fujisawa Y, Nakahashi S, Mizushina K, et al. Concordance between joint symptom/tenderness/ swelling and ultrasonography (US) synovitis in rheumatoid arthritis: Which clinical finding is more or less relevant to us synovitis than others? Annals of the Rheumatic Diseases. 2015;74:243. | Duplicate, poster or conference abstract only |
| 38. | Hirata A, Ogura T, Hayashi N, Takenaka S, Ito H, Mizushina K, et al. Concordance of Patient-Reported Joint Symptoms, Physician-Examined Arthritic Signs, and Ultrasound-Detected Synovitis in Rheumatoid Arthritis. Arthritis Care Res (Hoboken). 2017;69(6):801-6. | Not population of interest |
| 39. | Hirata A, Ogura T, Hayashi N, Yamashita N, Mizushina K, Nakahashi S, et al. Concordance between joint symptoms and ultrasonography findings in patients with rheumatoid arthritis. Annals of the Rheumatic Diseases. 2014;73. | Duplicate, poster or conference abstract only |
| 40. | Hirata A, Ogura T, Takenaka S, Ito H, Fujisawa Y, Mizushina K, et al. Concordance between ultrasound joint synovitis and clinical joint assessments by patients or physicians in rheumatoid arthritis. Arthritis and Rheumatology. 2016;68:710. | Duplicate, poster or conference abstract only |
| 41. | Hodge DK, Beaulieu CF, Thabit IGH, Gold GE, Bergman AG, Butts K, et al. Dynamic MR imaging and stress testing in glenohumeral instability: Comparison with normal shoulders and clinical/surgical findings. Journal of Magnetic Resonance Imaging. 2001;13(5):748-56. | No outcomes of interest |
| 42. | Huang LF, Rubin DA, Britton CA. Greater tuberosity changes as revealed by radiography: lack of clinical usefulness in patients with rotator cuff disease. AJR Am J Roentgenol. 1999;172(5):1381-8. | Not population of interest |
| 43. | Iorgoveanu VC, Bojinca V, Malitchi R, Gheorghe M, Ionescu C, Ionescu R. Shoulder ultrasonography in diabetic patients - is there damage with no clinical sign? Annals of the Rheumatic Diseases. 2017;76:1429. | Duplicate, poster or conference abstract only |
| 44. | Ishigaki T, Yoshino K, Hirokawa M, Sugawara M, Yamanaka M. Supraspinatus tendon thickness and subacromial impingement characteristics in younger and older adults. BMC Musculoskeletal Disorders. 2022; 23(1):1-9. | No outcomes of interest |
| 45. | Jigami H, Wada T, Matsumoto T, Koyama M, Kato T, Ohuchi H, et al. Characteristics of Masters swimmer's rotator cuff. Journal of Science and Medicine in Sport. 2017;20:2-3. | Duplicate, poster or conference abstract only |
| 46. | Jo W, Park HJ, Kim JN, Kim MS, Shin H, Kang CH. Aponeurotic expansion of the supraspinatus tendon and concomitant shoulder pathologies. European Radiology. 2023 Jan 16; 16:16. | No outcomes of interest |
| 47. | Johansson FR, Skillgate E, Adolfsson A, Jenner G, DeBri E, Swärdh L, et al. Asymptomatic Elite Adolescent Tennis Players' Signs of Tendinosis in Their Dominant Shoulder Compared With Their Nondominant Shoulder. Journal of athletic training. 2015;50(12):1299-305. | Not population of interest |
| 48. | Jost B, Zumstein M, Pfirrmann CWA, Zanetti M, Gerber C, Jost B, et al. MRI findings in throwing shoulders: abnormalities in professional handball players. Clinical Orthopaedics & Related Research. 2005;2005(434):130-7. | Duplicate, poster or conference abstract only |
| 49. | Karcich J, Kazam JK, Rasiej MJ, Wong TT. Bennett lesions in overhead athletes and associated shoulder abnormalities on MRI. Skeletal Radiol. 2019;48(8):1233-40. | Not population of interest |
| 50. | Kim J, Kim MW, Oh JH, Oh SJ. Ultrasonographic findings of shoulder disorders in breast cancer patients with lymphedema: Preliminary study. PM and R. 2010;2(9):S122. | Duplicate, poster or conference abstract only |
| 51. | Kohut K, Sabzevari S, Naendrup JH, Lin A. Acute proximal long head biceps rupture as a predictor of rotator cuff tears. Journal of Orthopaedic Research. 2017;35. | Duplicate, poster or conference abstract only |
| 52. | Krief OP, Huguet D. Shoulder pain and disability: comparison with MR findings. AJR Am J Roentgenol. 2006;186(5):1234-9. | Not population of interest |
| 53. | Kvalvaag E, Anvar M, Karlberg AC, Brox JI, Engebretsen KB, Soberg HL, et al. Shoulder MRI features with clinical correlations in subacromial pain syndrome: a cross-sectional and prognostic study. Bmc Musculoskeletal Disorders. 2017;18. | Not population of interest |
| 54. | Lee CS, Stetson WB, Goldhaber NH, Davis SM, Brock A, Wosmek J. Magnetic resonance imaging findings in asymptomatic elite volleyball players. Arthroscopy - Journal of Arthroscopic and Related Surgery. 2017;33(10):e58-e9. | Duplicate, poster or conference abstract only |
| 55. | Lee S-J, Tak S, Alterman T, Calvert GM. Prevalence of Musculoskeletal Symptoms Among Agricultural Workers in the United States: An Analysis of the National Health Interview Survey, 2004–2008. Journal of Agromedicine. 2014;19(3):268-80. | Not a study of the prevalence of imaging abnormalities |
| 56. | Lee SJ, Tak S, Alterman T, Calvert GM. Prevalence of musculoskeletal symptoms among agricultural workers in the United States: an analysis of the National Health Interview Survey, 2004-2008. Journal of Agromedicine. 2014;19(3):268-80. | Duplicate, poster or conference abstract only |
| 57. | Lim KB, Lee HJ, Joo SJ, Cho YJ, Chai JW. The Findings of Physical Examination and Ultrasonography at the Shoulders in High School Baseball Players. Annals of Rehabilitation Medicine-Arm. 2005;29(1):81-6. | No outcomes of interest |
| 58. | Liu R, Onks C, Thomas J, Walker E, Silvis M, Black K, et al. Prevalence of shoulder labrum abnormalities on MRI in a non-athletic asymptomatic young adult cohort. Clinical Journal of Sport Medicine. 2020;30(2):130. | Duplicate, poster or conference abstract only |
| 59. | Mascia AT, Salonen DC, Becker EJ, Miniaci A. MR imaging of the shoulder: Findings in asymptomatic professional baseball pitchers. Radiology. 1998;209P:499-. | Duplicate, poster or conference abstract only |
| 60. | Milgrom C, Schaffler M, Gilbert S, Vanholsbeeck M. ROTATOR-CUFF CHANGES IN ASYMPTOMATIC ADULTS - THE EFFECT OF AGE, HAND DOMINANCE AND GENDER. Journal of Bone and Joint Surgery-British Volume. 1995;77B(2):296-8. | Duplicate, poster or conference abstract only |
| 61. | Mirowitz SA. Normal rotator cuff: MR imaging with conventional and fat-suppression techniques. Radiology. 1991;180(3):735-40. | No outcomes of interest |
| 62. | Moeda F, Melo X, Hatia M, Pinho S, Calado D, de Andrade MR, et al. Clinical and ultrasound findings of 'swimmer's shoulder' and its association with training history in elite Portuguese swimmers: a cross-sectional study. Physician & Sportsmedicine. 2023 May 15:1-13. | Not population of interest |
| 63. | Moosmayer S, Tariq R, Stiris M, Smith HJ. The Natural History of Asymptomatic Rotator Cuff Tears A Three-Year Follow-up of Fifty Cases. Journal of Bone and Joint Surgery-American Volume. 2013;95A(14):1249-55. | Not a study of the prevalence of imaging abnormalities |
| 64. | Naranjo A, Marrero-Pulido T, Ojeda S, Francisco F, Erausquin C, Rúa-Figueroa I, et al. Abnormal sonographic findings in the asymptomatic arthritic shoulder. Scandinavian Journal of Rheumatology. 2002;31(1):17-21. | Not population of interest |
| 65. | Okada E, Matsumoto M, Ichihara D, Chiba K, Toyama Y, Fujiwara H, et al. Development of stiff shoulder in asymptomatic volunteers during ten-year follow-up in Japan. J Back Musculoskeletal Rehabil. 2010;23(2):69-75. | No outcomes of interest |
| 66. | Park HB, Gwark JY, Na JB. Risk factors of chronic subscapularis tendon tear. Clinics in Shoulder & Elbow. 2022 Dec; 25(4):257-264. | No symptom status available |
| 67. | Pennington RG, Bottomley NJ, Neen D, Brownlow HC. Radiological features of osteoarthritis of the acromiclavicular joint and its association with clinical symptoms. J. 2008;16(3):300-2. | Not population of interest |
| 68. | Pennock AT, Dwek J, Levy E, Stearns P, Manning J, Dennis MM, et al. Shoulder MRI Abnormalities in Asymptomatic Little League Baseball Players. Orthop. 2018;6(2):2325967118756825. | Not population of interest |
| 69. | Pope TL, Jr. Abnormal findings on magnetic resonance images of asymptomatic shoulders. J Bone Joint Surg Am. 1996;78(4):633-5. | Not a study of the prevalence of imaging abnormalities |
| 70. | Rajagopalan D, Abdelaziz A, Ring D, Slette E, Fatehi A. MRI findings of acromioclavicular joint osteoarthritis are the norm after age 40. Orthopaedics & traumatology, surgery & research. 2023 06; 109(4):103526. | No symptom status available |
| 71. | Ribeiro LP, Tazawa BMD, Barreto RPG, Camargo PR. Scapular kinematics and pathoanatomic findings: Are there differences between symptomatic and asymptomatic shoulders? Proceedings of Pain Science in Motion International and Interdisciplinary Colloquium on Research Methods in Pain Sciences - 4th edition, May 19-21, 2022, Maastricht, Netherlands. Pain Practice. 2022;22:14-. | Not a study of the prevalence of imaging abnormalities |
| 72. | Rossano A, Manohar N, Veenendaal WJ, van den Bekerom MPJ, Ring D, Fatehi A. Prevalence of acromioclavicular joint osteoarthritis in people not seeking care: A systematic review. Journal of Orthopaedics. 2022; 32:85-91. | Not a study of the prevalence of imaging abnormalities |
| 73. | Rowan KR, Andrews G, Spielmann A, Leith J, Forster BB. MR shoulder arthrography in patients younger than 40 years of age: frequency of rotator cuff tear versus labroligamentous pathology.[Erratum appears in Australas Radiol. 2007;51(4):402 Note: Forster, B B [added]]. Australasian Radiology. 2007;51(3):257-9. | Not population of interest |
| 74. | Salvarani C, Cantini F, Olivieri I, Barozzi L, Macchioni L, Niccoli L, et al. Proximal bursitis in active polymyalgia rheumatica. Annals of Internal Medicine. 1997;127(1):27-31. | Not population of interest |
| 75. | Samuel AM, Jain HM. Scintigraphic changes of osteoarthritis: An analysis of findings during routine bone scans to evaluate the incidence in an Indian population. Indian Journal of Nuclear Medicine. 2012;27(2):73-80. | Not a study of the prevalence of imaging abnormalities |
| 76. | Sell S, Zacher J, Konig S, Goethe S. Sonography in inflammatory rheumatic joint diseases. Ultraschall in der Medizin. 1993;14(2):63-7. | Not population of interest |
| 77. | Shaffer B, Huttman D. Rotator cuff tears in the throwing athlete. Sports Medicine and Arthroscopy Review. 2014;22(2):101-9. | Not a study of the prevalence of imaging abnormalities |
| 78. | Suh YS, Cheon YH, Kim HO, Lee WS, Yoo WH, Lim HS, et al. High prevalence of various upper limb musculoskeletal disorders in Korean orchardists. Annals of the Rheumatic Diseases. 2015;74:1301. | Duplicate, poster or conference abstract only |
| 79. | Tallay A, Pavlik A. Late effects of handball in female nationalteam players: High prevalence of pain, functional limitations and osteoarthritis ten years after winning the silver medal at the sydney olympic games. Arthroscopy - Journal of Arthroscopic and Related Surgery. 2011;27(10):e116-e7. | Duplicate, poster or conference abstract only |
| 80. | Tasaki A, Nozaki T, Morita W, Kobayashi D, Phillips BB, Kitamura N. The relationship between high-signal intensity changes in the glenohumeral joint capsule on MRI and clinical shoulder symptoms. Asiapacific Journal of Sports Medicine Arthroscopy Rehabilitation & Technology. 2020 Oct; 22:27-33. | Not population of interest |
| 81. | Toritsuka Y, Nakagawa S, Koyanagi M, Sado J, Nakata K, Masatomi T, et al. Shoulder and elbow evaluation of pitchers in National High School Baseball Invitational Tournaments and National High School Baseball Championships from 1993 to 2016 in Japan. Journal of Orthopaedic Science. 2020; 25(3):423-427. | No outcomes of interest |
| 82. | Turk AC, Fidan N, Ozcan O, Ozkurt S, Musmul A, Sahin F. Comparison of shoulder Magnetic Resonance Imaging findings between patients with stage 4 chronic kidney disease and hemodialysis patients with healthy controls. J Back Musculoskeletal Rehabil. 2020;33(2):179-84. | No outcomes of interest |
| 83. | Ueno A, Hirata M, Yamamura Y, Fujita K, Shibutou N, Yamamura M. Identification of risk factors for recurrence in polymyalgia rheumatica. Annals of the Rheumatic Diseases. 2018;77:1469. | Duplicate, poster or conference abstract only |
| 84. | Vahedi H, Fleischman AN, Salvo JP, Parvizi J. Higher Prevalence of Concomitant Shoulder Labral Tears in Patients With Femoroacetabular Impingement. Arthroscopy - Journal of Arthroscopic and Related Surgery. 2019;35(4):1074-9.e1. | Not population of interest |
| 85. | Wang JC, Shapiro MS. Changes in acromial morphology with age. J Shoulder Elbow Surg. 1997;6(1):55-9. | Not a study of the prevalence of imaging abnormalities |
| 86. | Williams PN, Calcei JG, Kontaxis A, Hillstrom H, Gulotta LV. Scapular kinematics in asymptomatic rotator cuff tears and healthy controls. Journal of Shoulder and Elbow Surgery. 2018;27(4):e124-e5. | Duplicate, poster or conference abstract only |
| 87. | Wright RW, Paletta Jr GA. Prevalence of the Bennett Lesion of the Shoulder in Major League Pitchers. American Journal of Sports Medicine. 2004;32(1):121-4. | No outcomes of interest |
| 88. | Wulff Svendsen S, Gelineck J, Egund N, Frost P. 0215Acromioclavicular joint degeneration in relation to cumulative occupational mechanical exposures: a magnetic resonance imaging study. Occupational & Environmental Medicine. 2014;71:A28-A. | Duplicate, poster or conference abstract only |
| 89. | Yamamoto A, Takagishi K, Kobayashi T, Shitara H, Ichinose T, Takasawa E, et al. The impact of faulty posture on rotator cuff tears with and without symptoms. J Shoulder Elbow Surg. 2015;24(3):446-52. | Not a study of the prevalence of imaging abnormalities |
| 90. | Yamamoto A, Takagishi K, Kobayashi T, Shitara H, Osawa T. Factors involved in the presence of symptoms associated with rotator cuff tears: a comparison of asymptomatic and symptomatic rotator cuff tears in the general population. J Shoulder Elbow Surg. 2011;20(7):1133-7. | Not a study of the prevalence of imaging abnormalities |
| 91. | Yoo YS, Park JY, Kim MS, Cho NS, Lee YB, Cho SH, et al. Calcific tendinitis of the shoulder in the Korean population: demographics and its relation with coexisting rotator cuff tear. Clinics in Shoulder & Elbow. 2021 Mar; 24(1):21-26. | Not population of interest |
| 92. | Yoon JP, Chung SW, Lee BJ, Kim HS, Yi JH, Lee HJ, et al. Correlations of magnetic resonance imaging findings with clinical symptom severity and prognosis of frozen shoulder. Knee Surg Sports Traumatol Arthrosc. 2017;25(10):3242-50. | Not population of interest |
| 93. | Zanetti M, Jost B, Hodler J, Gerber C. MR imaging after rotator cuff repair: full-thickness defects and bursitis-like subacromial abnormalities in asymptomatic subjects. Skeletal Radiol. 2000;29(6):314-9. | Not population of interest |

# Supplementary Table 7 – Included studies with unusable prevalence data

| 1. | Akbar M, Balean G, Brunner M, Seyler TM, Bruckner T, Munzinger J, et al. Prevalence of Rotator Cuff Tear in Paraplegic Patients Compared with Controls. Journal of Bone and Joint Surgery-American Volume. 2010;92A(1):23-30. | MRI | No prevalence data by shoulder symptom status for able bodied volunteer group. Requested but no response. |
| --- | --- | --- | --- |
| 2. | Akbar M, Brunner M, Ewerbeck V, Wiedenhöfer B, Grieser T, Bruckner T, et al. Do overhead sports increase risk for rotator cuff tears in wheelchair users? Archives of Physical Medicine and Rehabilitation. 2015;96(3):484-8. | MRI | No prevalence data by shoulder symptom status. Requested but no response. |
| 3. | Akbar M, Brunner M, Balean G, Grieser T, Bruckner T, Loew M, et al. A cross-sectional study of demographic and morphologic features of rotator cuff disease in paraplegic patients. Journal of Shoulder and Elbow Surgery. 2011;20(7):1108-13. | MRI | No prevalence data by shoulder symptom status. Requested but no response. |
| 4. | Boninger ML, Towers JD, Cooper RA, Dicianno BE, Munin MC. Shoulder imaging abnormalities in individuals with paraplegia. Journal of Rehabilitation Research and Development. 2001;38(4):401-8. | MRI | No prevalence data by shoulder symptom status. Email undeliverable. |
| 5. | Bovenzi M, Fiorito A, Volpe C. Bone and joint disorders in the upper extremities of chipping and grinding operators. Int Arch Occup Environ Health. 1987;59(2):189-98. | XRAY | No prevalence data by shoulder symptom status. |
| 6. | De Carli A, Mossa L, Larciprete M Ferretti M, Argento G, Ferretti A. The gymnast's shoulder MRI and clinical findings. Journal of Sports Medicine & Physical Fitness. 2012;52(1):71-9. | MRI | No prevalence data by shoulder symptom status. Requested but no response. |
| 7. | Fehringer EV, Sun JF, VanOeveren LS, Keller BK, Matsen FA. Full-thickness rotator cuff tear prevalence and correlation with function and co-morbidities in patients sixty-five years and older. Journal of Shoulder and Elbow Surgery. 2008;17(6):881-5. | US | No prevalence data by shoulder symptom status. Requested but no response. |
| 8. | Galluccio F, Bellucci E, Porta F, Tofani L, De Paulis A, Bianchedi D, et al. The waterpolo shoulder paradigm: results of ultrasound surveillance at poolside. BMJ Open Sport Exerc Med. 2017;3(1):e000211. | US | No prevalence data by shoulder symptom status. Requested but no response. |
| 9. | Gumina S, Candela V, Mariani L, Venditto T, Catalano C, Castellano S, et al. Rotator cuff degeneration of the healthy shoulder in patients with unilateral arm amputation is not worsened by overuse. Knee Surg Sports Traumatol Arthrosc. 2018;26(1):182-7. | MRI | No prevalence data by shoulder symptom status. Requested but no response |
| 10. | Hagemann G, Rijke AM, Mars M. Shoulder pathoanatomy in marathon kayakers. BJSM online. 2004;38(4):413-7. | MRI | No prevalence data by shoulder symptom status. Requested but no response. |
| 11. | Harada, Y., Yokoya, S., Sumimoto, Y., Iwahori, Y., Kajita, Y., Deie, M.,  & Adachi, N. (2022). Prevalence of Rotator Cuff Tears Among Older  Tennis Players and Its Impact on Clinical Findings and Shoulder Function. Journal of Sport Rehabilitation, 31(7), 849-855. | US | No useable prevalence data. Shoulder symptom status unclear. |
| 12. | Hirano Y, Sashi R, Izumi J, Itoi E, Watarai J. Comparison of the MR findings on indirect MR arthrography in patients with rotator cuff tears with and without symptoms. Radiat Med. 2006;24(1):23-7. | MRI | No prevalence data available by shoulder symptom status. No author contact details available. |
| 13. | Hodgson RJ, O'Connor PJ, Hensor EMA, Barron D, Robinson P. Contrast-enhanced MRI of the subdeltoid, subacromial bursa in painful and painless rotator cuff tears. British Journal of Radiology. 2012;85(1019):1482-7. | US | Number of imaged asymptomatic shoulders unclear. Requested but no response. |
| 14. | Holt K, Delbridge A, Josey L, Dhupelia S, Livingston GC, Jr., Waddington G, Boettcher C. Subscapularis tendinopathy is highly prevalent in elite swimmer's shoulders: an MRI study. Journal of Science & Medicine in Sport. 2022 Sep; 25(9):720-725. | MRI | No prevalence data by shoulder symptom status. Requested but no response. |
| 15. | Klein M, Tarantino I, Warschkow R, Berger CJ, Zdravkovic V, Jost B, et al. Specific Shoulder Pathoanatomy in Semiprofessional Water Polo Players: A Magnetic Resonance Imaging Study. Orthop. 2014;2(5):2325967114531213. | MRI | No prevalence data available by shoulder status for study groups. Requested but no response. |
| 16. | Lajtai G, Pfirrmann CWA, Aitzetmuller G, Pirkl C, Gerber C, Jost B. The Shoulders of Professional Beach Volleyball Players High Prevalence of Infraspinatus Muscle Atrophy. American Journal of Sports Medicine. 2009;37(7):1375-83. | US | No prevalence data by shoulder symptom status. Requested but no response. |
| 17. | Lee JC, Sykes C, Saifuddin A, Connell D. Adhesive capsulitis: sonographic changes in the rotator cuff interval with arthroscopic correlation. Skeletal Radiol. 2005;34(9):522-7. | US | No prevalence data for asymptomatic study group. Requested but no response. |
| 18. | Meroni R, Scelsi M, Boria P, Sansone V. Shoulder disorders in female working-age population: A cross sectional study. BMC Musculoskeletal Disorders. 2014;15(1). | US | No prevalence data by shoulder symptom status for study groups. Requested but no response. |
| 19. | Minagawa H, Yamamoto N, Abe H, Fukuda M, Seki N, Kikuchi K, et al. Prevalence of symptomatic and asymptomatic rotator cuff tears in the general population: From mass-screening in one village. J. 2013;10(1):8-12. | US | No prevalence data by shoulder symptom status. Author provided but not useable. |
| 20. | Monteleone G, Tramontana A, Mc Donald K, Sorge R, Tiloca A, Foti C. Ultrasonographic evaluation of the shoulder in elite Italian beach volleyball players. Journal of Sports Medicine and Physical Fitness. 2015;55(10):1193-9. | US | No prevalence data by shoulder symptom status. |
| 21. | Oh JH, Chung SW, Oh CH, Kim SH, Park SJ, Kim KW, Park JH, Lee SB, Lee JJ. The prevalence of shoulder osteoarthritis in the elderly Korean population: association with risk factors and function. J Shoulder Elbow Surg. 2011 Jul;20(5):756-63. | XRAY | No prevalence data by shoulder symptom status. Requested but not available. |
| 22. | Pearsall IAW, Bonsell S, Heitman RJ, Helms CA, Osbahr D, Speer KP. Radiographic findings associated with symptomatic rotator cuff tears. Journal of Shoulder and Elbow Surgery. 2003;12(2):122-7. | XRAY | No prevalence data by shoulder. |
| 23. | Pepke W, Brunner M, Abel R, Almansour H, Gerner HJ, Hug A, et al. [Risk factors for the development of rotator cuff tears in individuals with paraplegia : A cross-sectional study]. Orthopade. 2018;47(7):561-6. | MRI | No prevalence data by shoulder symptom status. Requested but no response. |
| 24. | Ro KH, Park JH, Lee SH, Song DI, Jeong HJ, Jeong WK. Status of the contralateral rotator cuff in patients undergoing rotator cuff repair. American Journal of Sports Medicine. 2015;43(5):1091-8. | US | No prevalence data by shoulder symptom status. Requested but no response. |
| 25. | Rodeo SA, Nguyen JT, Cavanaugh JT, Patel Y, Adler RS. Clinical and Ultrasonographic Evaluations of the Shoulders of Elite Swimmers. American Journal of Sports Medicine. 2016;44(12):3214-21. | US | No prevalence data by shoulder symptom status. Requested but no response. |
| 26. | Sansone V, Bonora C, Boria P, Meroni R. Women performing repetitive work: Is there a difference in the prevalence of shoulder pain and pathology in supermarket cashiers compared to the general female population? International Journal of Occupational Medicine and Environmental Health. 2014;27(5):722-35. | US | No prevalence data by shoulder symptom status for study groups. Requested but no response. |
| 27. | Scavenius M, Iversen BF. Nontraumatic clavicular osteolysis in weight lifters. American Journal of Sports Medicine. 1992;20(4):463-7. | XRAY | No prevalence data by shoulder symptom status. |
| 28. | Schar MO, Dellenbach S, Pfirrmann CW, Raniga S, Jost B, Zumstein MA. Many Shoulder MRI Findings in Elite Professional Throwing Athletes Resolve After Retirement: A Clinical and Radiographic Study. Clin Orthop. 2018;476(3):620-31. | MRI | No prevalence data by shoulder symptom status for former athletes. Requested but no response. |
| 29. | Toritsuka Y, Nakagawa S, Koyanagi M, Sado J, Nakata K, Masatomi T, et al. Shoulder and elbow evaluation of pitchers in National High School Baseball Invitational Tournaments and National High School Baseball Championships from 1993 to 2016 in Japan. Journal of Orthopaedic Science. 2020;25(3):423-7. | XRAY | No prevalence data per shoulder reported. |
| 30. | Yamaguchi K, Ditsios K, Middleton WD, Hildebolt CF, Galatz LM, Teefey SA. The demographic and morphological features of rotator cuff disease - A comparison of asymptomatic and symptomatic shoulders. Journal of Bone and Joint Surgery-American Volume. 2006;88A(8):1699-704. | US | No prevalence data by shoulder symptom status. Author advised requested data not available. |

# Supplementary Table 8 – Details on study population, participant recruitment, and outcomes

| **Details on study population, participant recruitment, and outcomes** | | | |
| --- | --- | --- | --- |
| **Study and location** | **Study population** | **Details of participant recruitment** | **Outcome definition** |
| **X-ray and MRI studies** | | | |
| **Gill et al 2014**  Australia | Population-based | Participants were obtained from the North West Adelaide Study (NWAHS), a longitudinal cohort study of 4056 randomly selected adults aged 18 years and over at the time of recruitment from the northern and western regions of Adelaide, South Australia. This sample region represents approximately half of the metropolitan area of Adelaide (total population of approximately 1.2 million) The study ran from 1999 to 2003 with Stage 1, Stage 2 was conducted between 2004 and 2006 and Stage 3 was conducted between 2008 and 2010, with the aim of providing longitudinal measured and self-reported data.  Potential participants for this study were identified from the NWAHS database in the following manner. In Stage 2, participants were asked: ‘Have you ever had pain or aching in your shoulder, either at rest or when moving, on most days for at least a month?’ In Stage 3, participants were asked: ‘Over the past month, have you had pain or aching in either or both of your shoulders, either at rest or when moving, on most days?’ Respondents who replied in the negative to both questions were identified as those with no current or previous shoulder pain; those who responded in the affirmative to the shoulder pain question in Stage 2 were those with previous shoulder pain and those who responded ‘yes’ to both questions were those with current shoulder pain. Participants were excluded if they were not currently aged between 55 and 74 years and if they self-reported that they had doctor-diagnosed rheumatoid arthritis in either Stage 2 or Stage 3. | Both the X-ray and MRI films were independently read by two experienced musculoskeletal radiologists blinded to each participant’s symptoms.  The **X-ray findings** are summarized in Table 2. These findings and subsequent categorizations were the subjective judgement of the reporting radiologists and based on their normal clinical practice and experience. Any discrepancy between the two radiologists was discussed and decided upon by consensus.  **MRIs** were read using a structured reporting system. As there are no validated scoring systems for MRI scans of the shoulder, subjective judgement and clinical experience were used to determine the following:  **ACJ arthritis** severity which was determined according to the degree of osteophytes, joint effusion, synovial thickening, bone edema and articular cartilage thinning;  **Subacromial bursitis**: mild bursitis had a sliver of fluid present or a small increase in T2 signal; moderate bursitis, clear fluid or thickening present; and severe bursitis, marked fluid distension and synovial thickening and/or the presence of rice bodies;  *No specific classifications.* |
| **X-ray studies** | | | |
| **Worland et al 2003**  USA | Miscellaneous (volunteers) | Between November 1999 and January 2000, the shoulders of 59 asymptomatic volunteer subjects were studied. The authors performed 18 outlet x-ray views and 18 shoulder rotator cuff ultrasound scans. Al volunteers had no past history of shoulder injury or surgery and were completely asymptomatic with regard ot their shoulders. The subjects were divided into four groups by age. Group 1 consisted of subjects 40-49 years of age (seven males, eight females); group ,2 50-59 years of age (seven males, eight females); group ,3 60-69 years of age (eight males, six females); and group 4 over 07 years (seven males, eight females). Al patients underwent a clinical evaluation that showed no functional alteration in terms of range of movement, power, or impingement signs. | The evaluation of the **acromion type** was performed by drawing the acromial angle as described by Toivonen. The angle is formed by the intersection of a line along the undersurface of the acromion and a line extending from the tip of the hook to a point where the base of the hook joins the undersurface of the acromion. Two independent readings were performed by the authors. The acromial type was defined by the value of the acromial angle. Type I acromions had an acromial angle of °0 - 12°; type I had 13° 2-7°; and type I had greater than 27°. |
| **Maquirriain et al 2006**  Argentina | Miscellaneous (volunteers) | We studied 18 asymptomatic senior tennis players (17 male; mean (SD) age, 57.2 (8.8) years, range 51 to 75) and 18 matched controls (17 male; 59.8 (6.4) years, range 51 to 76).  Inclusion criteria for the control group were: age older than 50 years; no history of shoulder surgery, trauma, or systemic arthropathy; and sedentary habits and lack of heavy working tasks. | Images were analysed by two experienced musculoskeletal radiologists. All evaluations were made with the radiologists blinded to the age and sex of the subjects. Acromioclavicular joint degeneration, subacromial calcification, and superior migration of the humeral head were also recorded.  *Definition not specified.* |
| **Khoschnau et al 2020**  Sweden | Miscellaneous (healthcare population and volunteers) | Between September 2007 and December 2009, subjects for this study were recruited through a questionnaire that was given to patients and the relatives who accompanied them when they sought our hospital outpatient clinics for urological, gynecological or ear-nose-throat conditions. Subjects seeking for orthopedic problems were excluded from this study.  All subjects aged 50–75 (median 66 years) were contacted and asked to participate in this research project. The participation was voluntary and without cost for the subjects. | Bilateral ultrasound examination was performed by one examiner. A radiological examination of the shoulders was also performed, and it included standard anteroposterior views with the glenoid in absolute profile and the head of the humerus in neutral position. A standardized shoulder radiographic protocol listed different areas to be examined.  *Definition not specified.* |
| **Maquirriain et al 2006**  Argentina | Athletes  (former elite tennis players) | We studied 18 asymptomatic senior tennis players (17 male; mean (SD) age, 57.2 (8.8) years, range 51 to 75) and 18 matched controls (17 male; 59.8 (6.4) years, range 51 to 76).  Inclusion criteria for the study group were: age older than 50 years; previous professional level and continuous activity (practising, teaching); no history of surgery or major trauma (that is, fracture or dislocation) to either shoulder; and no history of systemic arthropathy. They had begun playing the sport at a mean age of 8.0 (2.6) years. | Images were analysed by two experienced musculoskeletal radiologists. All evaluations were made with the radiologists blinded to the age and sex of the subjects. **Acromioclavicular joint degeneration**, subacromial **calcification**, and superior migration of the humeral head were also recorded.  *Definition not specified.* |
| **Wright et al 2007**  USA | Athletes  (overhead, baseball pitchers) | Fifty-seven asymptomatic MLB pitchers participating in the St Louis Cardinals spring training camp underwent routine preseason radiographic screening of their dominant shoulder and elbow between 1986 and 1998. Radiographs were retrospectively reviewed. | The radiographs were reviewed by a single investigator, and no intraobserver reliability was performed. The radiographic changes reviewed in this study are not represented by classification systems in the literature, and thus severity of findings cannot be presented in a standardized fashion. Thus, the authors chose to use a system of present or not present.  *Definition not specified.* |
| **Ultrasound studies** | | | |
| **Wang et al 2005**  Taiwan | Miscellaneous (volunteers and athletes) | The inclusion criterion for the elite college baseball athletes sampled in this study was that they either played for the national baseball team during the World Cup in 2001, or trained in the preparatory team. Fifty-four elite college baseball athletes (mean age: 20) were recruited from among 75 eligible subjects. The recruitment rate of athletes thus was 72%. The baseball athletes who had suffered shoulder sports injuries or pain were identified as the group of injured athletes, while those athletes who reported no history of injury or pain comprised the group of uninjured athletes. Meanwhile, the inclusion criteria for the control group were as follows: first, control group members were completely asymptomatic and had no history of shoulder pain or injury causing them to seek medical treatment or surgery. Second, control group members did not participate in sports at a professional level and were not studying physical education. Third, control group members were matched in physical characteristics with the recruited athletes. | **Acromioclavicular degenerative changes**: Cortical irregularities or osteophytes, usually accompanied by intraarticular hypoechoic fluid displacing joint capsule, i.e., effusion, or joint bulging 42 mm by longitudinal scanning (Naredo et al., 2002)  *No specific classification.* |
| **Oschman et al 2007**  South Africa | Miscellaneous (healthcare population) | The shoulders of 50 patients, 32 males and 18 females, with an average age of 64 years (range 40 - 83 years) were studied. The patients had a confirmed symptomatic rotator cuff tear on the one side and an asymptomatic shoulder on the contralateral side. The asymptomatic shoulder had no history of problems severe enough to have required medical attention. | The normal SASD bursa is not thicker than the humeral head cartilage and does not contain fluid. If the bursa contained fluid and appeared thickened it was documented as a **SASD bursitis** *(No specific classification)*.  Signs of **impingement** according to Neer’s classification and size of the tears. Stages of impingement on ultrasound according to Neer are as follows:  • Stage I - oedema and haemorrhage in the bursa and rotator cuff.  • Stage II - fibrosis and thickening of the bursa and partial rupture of the rotator cuff.  • Stage III - complete rupture of the rotator cuff. |
| **Abate et al 2010** Italy | Miscellaneous (healthcare population) | All the subjects enrolled in the study were recruited from the Outpatients Service of the Medicine and Science of Aging Department of Chieti - Pescara University. Inclusion criteria were the following: 1) living independently in the community; 2) age > 65 years; 3) right - handedness; 4) absence of pain or acceptable discomfort in the shoulder joint, spontaneous or during usual activities of daily living; 5) no subjective dysfunction; 6) no history of trauma or surgery of the shoulder joint. Patients with rheumatic disorders, endocrinopathies, malignancies and systemic diseases (renal, hepatic, cardiac, etc.), treated with steroids or NSAID, were excluded. The local Ethics Committee approved the study design and informed written consent was obtained from all the patients. The study group included 48 subjects with non-insulin-dependent diabetes mellitus (NIDDM). The diagnosis of NIDDM was based on American Diabetes Association criteria. The control group was made by 32 subjects, matched for age and sex, but without NIDDM, and selected with the same inclusion/exclusion criteria. | Involvement of **SAD** was identified when accumulation of anechoic fluid, with or without hypoechoic swelling of the synovia, appeared within it; it was graded subjectively as normal (distension < 1 mm), slightly increased (1 - 2 mm) or clearly increased (> 2 mm).  *No specific classification.* |
| **Ocguder et al 2010** Turkey | Miscellaneous (volunteers)  Athletes (overhead elite premier league) | The study evaluated the shoulders of overhead elite premier league athletes involved in basketball, handball, volleyball, body building, and water polo. The study protocol was approved by the institution- al ethics committee, and informed consent was obtained from all the subjects. Ultrasonographic examination of both shoulders was performed in 45 asymptomatic overhead athletes (8 female, 37 male ; mean age: 22 years, age range : 17-40 years), and 43 asymptomatic volunteers (13 female, 30 male ; mean age : 25 years, age range: 18-33 years). Although the age distribution was within a large range, all sportsmen were playing in elite premier league and were randomly selected from the group of four overhead sports mentioned above. The shoulders of all the overhead athletes and asymptomatic volunteers were completely asymptomatic, and the subjects had no history of previous shoulder injury, symptoms, and/or surgery. Each of the 45 players and 43 control subjects had an independent physical examination of both shoulders performed by an orthopaedic shoulder surgeon. Any current or former shoulder pain was recorded. All the shoulders were inspected for scars, atrophy, deformities and rhythm of motion. The rotator cuff was evaluated using Jobe’s test and impingement was evaluated with Hawkins and Yocum’s tests. | The thickness of the SS tendon was measured at 5mm and 10 mm from its humeral insertion. In addition, any tears, **calcifications**, and tendinitis were recorded.  **Subacromial–subdeltoid bursa** thickness between the folds of the bursa was measured. Any effusions were recorded.  *Definition not specified.* |
| **Girish et al 2011**  USA | Miscellaneous (healthcare population, males with knee problems) | The patient sample consisted of 51 consecutively registered men without symptoms who were recruited as a part of a wider study of bone density in the first half of 2009. These patients had gone to the orthopedic clinic because of knee problems. All subjects were men because women were excluded from the study to avoid postmenopausal effects on humeral bone density. All subjects included in the study reported no symptoms, trauma, or treatment involving either shoulder as determined by a sport medicine–trained orthopedic shoulder surgeon. No subject had a history of systemic inflammatory disease. Medical records, including reports of other imaging studies, were reviewed. | **Subacromial-subdeltoid bursal** thickening was defined as focal or diffuse bursal thickening of more than 2-mm transverse thickness with associated hypoechogenicity with or without bursal fluid.  **Subacromial impingement** was defined as pooling of subacromial-subdeltoid bursal content just lateral to the acromion at dynamic shoulder abduction evaluation.  **Acromioclavicular osteoarthritis** was defined as the presence of osteophytes with associated articular surface irregularity with or without joint effusion or capsular thickening.  *Definition not specified.* |
| **Iagnocco et al 2013**  Italy | Miscellaneous (volunteers from 4 Italian rheumatology units) | Ninety-seven healthy subjects were enrolled in the present study. The study was conducted in 4 Italian Rheumatology Units (Sapienza Università di Roma, Università Politecnica delle Marche, Università di Pisa and Università di Pavia). The presence of musculoskeletal symptoms, including painful shoulder, and a diagnosis of any rheumatic diseases as well as any other systemic pathology were exclusion criteria from the study. | In each of the 4 units participating in the study, US examination was separately and independently performed by a single ultra- sonographer who was a rheumatologist experienced in musculoskeletal US. The synovial structures of the shoulder, including the **subacromial/subdeltoid bursa** and the **ACJ** were examined for the presence of synovial effusions (SE) and synovial hypertrophy (SH). In addition, ACJ was analysed for the detection of osteophytes and erosions as well as of fibrocartilage calcifications. All abnormalities were studied according to international accepted definitions and scored according to a dichotomous assessment. In addition, calcifications were assessed for the presence of acoustic shadowing and their maximal dimension was measured.  *Definition not specified.* |
| **Sansone et al 2016**  Italy | Miscellaneous (healthcare population, females referred to routine gynecological screening) | Between January and March 2013 all the patients referred to a gynaecological clinic for routine screening were asked if they were willing to participate in our study. Inclusion criteria for our study included female gender and age between 18 and 60 years old. Exclusion criteria were current pregnancy, having had previous shoulder surgery or shoulder fractures, or a diagnosis of cancer. Three hundred and two female volunteers (604 shoulders) met the inclusion/exclusion criteria and agreed to participate. | The US was performed by a single consultant radiologist with more than 10 years of musculoskeletal scanning experience. Between the deltoid muscle and the external rotator tendons the **subacromial-subdeltoid bursa** was seen, appearing as a hypoechoic line of a thickness of less than 2 mm with a variable amount of peribursal echogenic fat (*No specific classification)*. Rotator cuff **calcifications** were defined as echogenic focus with or without posterior acoustic shadowing. The maximum diameter of the calcifications was measured, and they were classified as follows: less than 2 mm; between 2 and 5 mm; and greater than 5 mm.  *Calcification calssified according to size.* |
| **Meroni et al 2017**  Italy | Miscellaneous (volunteers, working aged women) | From November 2011 to March 2012, 305 female customers of a supermarket chain in northern Italy were recruited to participate in this study. A consecutive sampling technique was used to enroll the participants. All the subjects were aged between 19 and 56 years. The exclusion criteria were a previous shoulder trauma or surgery, evident or previously diagnosed major pathologies, and the presence of other musculoskeletal, neurological, or psychiatric impairments. Furthermore, subjects who performed repetitive movements of the upper arm or carried heavy loads for professional reasons were also excluded.  Respondents were asked if they had had any musculoskeletal disorders in the last 12 months that had prevented normal activity and if they had had shoulder pain at least once a month in the past year or during at least seven consecutive days in the past year, or if they felt continuous pain. A negative response was considered as an “asymptomatic shoulder”; otherwise, a positive response was considered as “symptomatic shoulder.” | The following structures were examined: rotator cuff (supraspinatus, infraspinatus/teres minor, and subscapularis), long head of the biceps tendon, **SAD bursa**, humeral head, and **acromioclavicular joint**. Tendon lesions, tendon calcifications, bursitis, capsulitis, and all other morphological and degenerative abnormalities such as arthritic alterations of the surrounding structures were registered. All abnormalities were studied according to the international accepted definitions and scored according to a dichotomous assessment.  *No specific classifications.*  **Calcifications** were assessed for the presence of acoustic shadowing and further divided into 3 groups (granular, milk, or linear calcifications), and were then further classed according to diameter size as follows: calcifications between 5 and 12 mm, and calcifications >12 mm in diameter.  *Calcification calssified according to size.* |
| **Suzuki et al 2021**  Japan | Miscellaneous (volunteers)  Athletes  (masters level swimmers) | A total of 60 subjects (23 men and 37 women) aged 33 to 65 years participated in this study; 40 were competitive swimmers and 20 were healthy sex- and age-matched controls. The common inclusion criteria were as follows: aged 30 to 65 years at the time of the survey and no history of shoulder fractures, shoulder dislocation, or shoulder surgery. In addition, swimmers included those who had practiced swimming regularly at least once a week in the preceding year, while controls were those who were not engaged in regular exercise or sports activities at enrolment. We excluded swimmers with less than three years of competitive swimming history and controls with shoulder pain in ADL at the time of the survey. | All ultrasonographic evaluations were performed by a physical therapist with more than 5 years of musculoskeletal scanning experience according to the technical guidelines of the Ultrasound Subcommittee of the European Society of Musculoskeletal Radiology. **Calcification** was defined as intra-tendinous hyperechoic areas with or without posterior acoustic shadowing.  The **SAB** was evaluated for the presence of thickening/effusion and defined as focal or diffuse bursal thickening of more than 2 mm. SAB thickness was measured from the top to the bottom at the first change point of the inclination angle  *No specific classifications.* |
| **Eliason et al 2022** Sweden | Miscellaneous (healthcare population) | Patients were consecutively recruited from seven primary care centers in Stockholm between 2012 and 2016. One hundred fifteen patients, 54 men and 61 women diagnosed with unilateral SAPS were included in the study. The patients were 20–59 years of age (mean 45, 0 ± 10, 4) with a pain duration of mean 22 ± 14.5 weeks.  Inclusion and Exclusion Criteria  The *inclusion criterion* was patients aged 20–59 years that had suffered from SAPS between 4 weeks and 1 year. We used the recommended combination of clinical tests for evaluating the patients. For inclusion at least two of these tests had to be positive together with a positive painful arc.  *Exclusion criteria* were bilateral shoulder pain, earlier treatment with cortisone injection, diabetes mellitus, history of trauma, fractures and dislocations of the shoulder joint, rheumatoid arthritis, severe arthroses, frozen shoulder (loss of passive and active range of motion), a positive drop-arm test and other clinical signs of total tears or FTT ́s. Fibromyalgia and thoracic or cervical spine syndromes. | The **acromioclavicular joint** was evaluated in terms of possible sprains and osteoarthritis. The supraspinatus tendon and the **sub- deltoid/subacromial bursae** were evaluated dynamically during abduction with the arm slightly internally rotated.  The US examiner was blinded to arm dominance, the patient ́s symptom as well as to the purpose of the study. The sonographic evaluation of the rotator cuff was performed in all patients according to a standardized protocol, the same that was thoroughly described by Vlychou et al. (2009).  *No specific classifications.* |
| **Brasseur et al 2004**  France | Athletes  (veteran tennis players) | In July 1998, the French Tennis Federation invited the 600 players participating in the French veteran championship of the Roland Garros Tennis Open to undergo free clinical and US examinations of their shoulders. The first 150 volunteers constituted the cohort of subjects used for this study. This cohort included 85 men (age range 35–76 years, mean age 57 years) and 65 women (age range 35–77 years, mean age 52 years). | An **effusion of subacromial–subdeltoid bursa (SSB)** was defined as an anechoic lamina between the folds of the bursa. It was considered to be mild (lamina thickness <3 mm) or abundant (lamina thickness >3 mm).  The **SSB thickness** was measured. The SSB was considered to be abnormal when the hypoechoic lamina located between the deep hyperechoic lamina of the deltoid and the superficial hyperechoic lamina of the supraspinatus was >2 mm thick. It was considered to be mildly thickened when this lamina was 2 or 3 mm, and markedly thickened when >3 mm.  *No specific classifications.* |

| **MRI studies** | | | |
| --- | --- | --- | --- |
| **Chandnani et al 1992**  USA | Miscellaneous (volunteers) | Twenty volunteers, who had never had symptoms referred to the shoulder, aged 25-55 years, were included | Images were blindly and independently interpreted by three radiologists experienced in skeletal MR and differences resolved by consensus.  The subacromial and subdeltoid region was evaluated for the presence of degenerative changes in the region of the **acromioclavicular (AC) joint**, and supraspinatus depression was sought.  *Definition not specified.* |
| **Neumann et al 1992**  USA | Miscellaneous (volunteers) | The study includes MR images of55 shoulders in 32 asymptomatic young, active, and healthy men and women in medical services or enlisted in the Armed Forces. The 23 men and nine women were 22- 45 years old (mean, 26 years). | The images were reviewed by radiologists experienced in musculoskeletal MR imaging. The **SA-SD space** was evaluated for presence, continuity, and signal intensity of the fat stripe or **fluid** in the bursa. When present, changes suggesting reactive thickening or osteophytes on the inferior cortex of overlying acromion were recorded, as were inferior osteophytes and other degenerative changes **of the acromioclavicular joint**.  *No specific classifications.* |
| **Needell et al 1996**  USA | Miscellaneous (volunteers from local community) | Volunteers were solicited by advertisement in the local community for participation in a sports medicine study. The purpose and method of the study were not made known. A detailed questionnaire covering occupational history, past medical history, handedness, and activity level was distributed to 153 respondents. Those with a history of shoulder or neck trauma, surgery, or pain were excluded from the study. The remaining 107 underwent clinical examination; seven had abnormal clinical examination findings (shoulder symptoms), and were excluded from the study, leaving 100 asymptomatic volunteers to participate. The final study population included 51 women and 49 men who were 19-88 years old (mean, 54 years old). | Bone changes associated with **impingement**, including AC OA, subacromial spurs, and humeral head cysts were evaluated. **Acromioclavicular joint osteoarthrosis** was subjectively quantified as mild, moderate, or severe. The diagnosis of **subacromial spurs** was made by observing the presence of a marrow signal or a signal void projecting from the acromion tip.  *No specific classifications.* |
| **Stein et al 2001**  USA | Miscellaneous (healthcare population, other musculoskeletal complaint) | MRI of the ACJ and surrounding structures was performed on 50 shoulders in 42 patients who ranged in age from 19 to 72 years (average, 35 years). There were 18 men and 24 women. Twenty-three right shoulders and 27 left shoulders were studied. There were 44 right-hand–dominant and 6 left-hand–dominant patients. MRI was performed on 21 dominant shoulders and 29 non- dominant shoulders. Patients who were having MRI of other extremities were asked to participate in this study. All patients were screened before participation with a standardized questionnaire and excluded if there was any history of shoulder pain, instability, decreased range of motion, trauma, infection, or arthridities. | Images were evaluated by an experienced musculoskeletal radiologist for the presence of ACJ abnormalities consistent with arthritis. All evaluations were made with the radiologist blinded to the age and sex of the patient. Determination of the grade of **ACJ abnormalities** was based on multiple factors including joint space narrowing, subchondral irregularity, capsular distension, and osteophyte formation. *Grade I* was characterized as having no capsular dis- tension, no joint space narrowing, and no evidence of osteophyte formation. Mild changes (*grade II*) were characterized by capsular distension, which was frequently the sole finding but was occasionally accompanied by mild joint space narrowing. Moderate changes (*grade III*) were demonstrated by capsular distention with a combination of joint space narrowing, subacromial fat effacement, and marginal osteophyte formation. Severe ACJ changes (*grade IV*) included all of the above in addition to marked joint space irregularity and narrowing with large osteophytes.  *ACOA Stein Classification* |
| **Barreto et al 2019**  Brazil | Miscellaneous (volunteers from the community who had self-reported unilateral shoulder pain) | Recruitment was performed by advertisements on local websites and printed flyers at the university and in the community. 347 eligible, 224 excluded  Individuals with atraumatic self-reported unilateral shoulder pain for at least 4 weeks since first onset and full active arm elevation | **Acromioclavicular (AC) joint** alterations such as osteoarthritis (OA) or joint hypertrophy were observed in the coronal oblique and sagittal oblique planes. Cysts and fluid at the AC joint were observed on T2 images. Signs of tissue proliferation such as osteophytes, joint space narrowing, margin irregularity, and bone sclerosis were identified on T1 images.  Increased **subacromial fluid** was reported when the subacromial bursa contained signal intensity equal to the signal of joint fluid or water on T2 images.  *No specific classifications.*  **Acromial morphology** was described following the classification of Epstein et al, divided into 3 types: type I, flat; type II, smoothly curved; and type III, hooked. |
| **Su et al 2020**  Taiwan | Miscellaneous (male volunteers)  Athletes  (male baseball players) | 30 age-matched male recreational athlete volunteers. These volunteers were radiologic technologists and general personnel at our institution.  We identified 76 male baseball players of the professional baseball league in Taiwan from records of MRI examinations performed between 2013 and 2015. | **SA-SD bursa:**  Edematous: Bursa with high signal intensity or apparent effusion on T2-weighted images.  Thickened: Bursa with a thickness greater than 2 mm.  *No specific classification.* |
| **Liu et al 2021** USA | Miscellaneous (non-athletic volunteers from a college and medical school) | Participants were recruited from a local college and medical school by way of flyers, Studyfinder, and social media. Inclusion criteria included age 18–29, willingness to undergo MRI of both shoulders, no current shoulder injury/complaints, and must not have ever participated in formal competitive athletics (including junior/varsity level high school, college, competitive club sports, or deemed self to have played any sports at an amateur/competitive level). Exclusion criteria included non-English speaking, age younger than 18 or older than 29, contraindication to MRI, previous shoulder surgery, and previous/current shoulder injuries. | The images were reviewed with data recorded on a standardized form evaluating the following areas: joint fluid, bone marrow signal, rotator cuff tendon, biceps tendon, labrum, acromioclavicular (AC) joint, and finally chondral injuries of the glenohumeral joint. Three months later, both radiologists blinded to their previous readings reviewed the MRIs for a second time, recording their findings on a new standardized form.  A given finding was considered positive for the epidemiologic prevalence if there was agreement in a specific anatomic area by the majority of the four reads (at least three positive reads out of a total of four—two reads at two different points in time).  *No specific classifications.* |
| **Miniaci et al 2002**  Canada | Athletes (professional male baseball pitchers) | Twenty-eight shoulders in 14 male professional baseball pitchers (average age, 20.1 years; range, 18 to 22) were evaluated in this study. Eleven of the 14 pitchers were right-handed and 3 were left-handed. Each pitcher had at least 1 year of professional experience. All athletes were completely asymptomatic with regard to their shoulders and had no history of previous shoulder injury, symptoms, or surgery.  Players were evaluated and underwent MR imaging at the Toronto Western Hospital in Toronto, Ontario, Canada. | The **acromial shape** was assessed in all 28 shoulders imaged on both the sagittal and coronal oblique views. The sagittal oblique view is best for determining acromial shape, although large spurs are readily identified on the coronal oblique view. The acromions were classified with respect to their appearance on the image just lateral to the acromioclavicular joint. In addition, the presence of **subacromial enthesophytes** and **acromioclavicular joint osteoarthritis** was noted.  *No specific classifications.* |
| **Connor et al 2003**  USA | Athletes (elite overhead athletes) | Prospective MRI investigation of dominant and nondominant shoulders of young, elite overhead athletes. Twenty elite overhead athletes (12 college baseball pitchers and 8 professional tennis players) participated in this investigation. The average age of the athletes was 26.4 years, and the average length of athletic participation was 16.7 years | Three musculoskeletal radiologists who were blinded to clinical data independently interpreted each MRI. Each musculoskeletal radiologist completed an image interpretation form for each scan, evaluating the outcome variables:  **Subacromial fluid** (absent/present)  **Acromiohumeral distance** (normal/abnormal)  **Calcification** (absent/present)  Articular cartilage (normal/abnormal)  Joint effusion (absent/present)  Labrum (normal/abnormal)  Biceps tendon (normal/torn)  Greater tuberosity (normal/sclerosis/cysts)  **Acromioclavicular joint** (normal/arthritic)  *No specific classifications.* |
| **Reuter et al 2008**  USA | Athletes (Ironman participants) | Each Ironman Triathlete underwent a comprehensive physical examination of the shoulder of the dominant arm by an experienced orthopedic surgeon (WDH). The findings from the physical examination in addition to initial screening by the research coordinator were used to ensure proper inclusion of the study subjects into their respective study groups. These two groups were defined as follows: *(1) asymptomatic Ironman Triathletes* [seven subjects, five men and two women; average age 35, range 29–62], *(2) symptomatic Ironman Triathletes* (16 subjects, 11 men and five women; ave. age 39, range 27–59). All 23 subjects (average age 37) were 18 years of age or older and participating as Ironman Triathlete competitors. | The **AC joint** was evaluated specifically for elevated bone marrow signal within the clavicle and acromion.  *No specific classifications.* |
| **Del Grande et al 2016**  USA | Athletes (male overhead athletes; dominant shoulders of 19 asymptomatic baseball pitcher draft picks) | We selected from our electronic patient record, 20 asymptomatic baseball pitcher draft picks that underwent 3-T MR examination of the shoulder between January 2008 to September 2013. All subjects underwent physical exam by an experienced orthopedic surgeon with special training in sports medicine within 2 day of the MRI examination. The physical examination included but was not limited to acromioclavicular and sternoclavicular tenderness, O'Brien test, Jobe relocation test, and subacromial impingement test. Exclusion criteria were: history of shoulder surgery, abnormal physical examination, or nondiagnostic image quality. One subject was excluded because of previous shoulder surgery. Therefore, the final study population included 19 asymptomatic baseball pitchers draft picks with a mean age of 19.9 years (range, 17–22 years). All subjects were men. | **Acromioclavicular joint**: Osteoarthritis was graded as none, mild (capsular distension), moderate (capsular distension, joint space narrowing and marginal osteophytes), or severe (capsular distension, joint space narrowing, joint space irregularity, and large osteophytes) following modified criteria used by Shubin Stein et al.  *Stein classification.*  **Subacromial space**: Fluid in the subacromial space was recorded as absent, linear (1 mm thickness), or saclike (greater than 1 mm thickness).  *No specific classification.* |
| **Celliers et al 2017**  South Africa | Athletes  (elite swimmers) | The study population was voluntarily selected from the University of the Free State’s swimming team located in Bloemfontein, South Africa. The sample included 20 volunteers, both male and female elite swimmers between the ages of 16 and 25 years, with symptomatic and asymptomatic shoulders. An elite swimmer was defined as a swimmer with the ability to perform a 100 m freestyle race at or faster than 75% of a national record swimming time. Exclusion criteria were: previous shoulder surgery, previous fracture of the shoulder, inability or unwillingness to participate in the MRI and clinical shoulder examination. None of the swimmers were excluded from the study. | The images were reviewed by three consultant radiologists and findings were documented on a **standardised self-administered assessment form** that was compiled from Stoller’s textbook of MRI in orthopaedics and sports medicine. The checklist consisted of 135 variables that had to be evaluated in the three different planes. For each item, a yes or no had to be indicated by the radiologists. For analysis, consensus between the radiologists was used (i.e. the answer given by two or more of the radiologists).  *No specific classifications.* |
| **Hacken et al 2019**  USA | Athletes (college and professional male ice hockey players) | Collegiate hockey players from a National Collegiate Athletic Association Division III team and professional hockey players from an American Hockey League/National Hockey League team were recruited for the study. Fifty shoulders in 25 male collegiate (n=13) and professional (n=12) ice hockey players were examined. To be eligible, players needed to be >18 years, active members of team and willing to undergo MRI. Athletes were excluded from the study if there was any current or previous known shoulder injury, as determined by player records and patient self-reporting | Images were blindly reviewed by 2 board-certified radiologists specialized in musculoskeletal radiology with extensive experience in reading shoulder MRIs. Data from the reviews were recorded on a standardized form evaluating the following 7 areas for each side: joint fluid, bone marrow signal, rotator cuff tendon, biceps tendon, superior/anterior/posterior/inferior labrum, **AC joint**, and determination of whether chondral injuries of the glenohumeral joint were present. Three months later, both radiologists, blinded to their previous readings, reviewed the MRIs for a second time and recorded their findings on a new standardized form. Thus, there were 4 interpretations for each MRI.  Regarding overall prevalence, a finding at a given shoulder site and side was considered positive for abnormality if at least 3 of the 4 interpretations (ie, >50%) were classified as positive; otherwise, a negative finding was recorded.  *No specific classifications.* |
| **Lee et al 2020**  USA | Athletes  (26 elite volleyball players from the US Men’s and Women’s National Indoor Volleyball Teams) | A total of 26 elite volleyball players from the US Men’s and Women’s National Indoor Volleyball Teams consented to participate in this study. Inclusion criteria for the study included playing nationally and internationally with no pain or restrictions to activity level at the time of the study (asymptomatic). Exclusion criteria included pain in the dominant shoulder of the athlete, having gone through a rehabilitation programme within the year before data collection, previous surgery on the dominant shoulder and regular use of pain or anti-inflammatory medications at the time of data collection (symptomatic). | Each athlete underwent a (non-funded) non-arthrogram MRI (Esaote S-Scan 0.27 Tesla).  MRI studies were reviewed by two radiologists with fellowship training in musculoskeletal radiology. Both radiologists were blinded. Data was only included when there was interobserver agreement between both radiologists. Data regarding the osseous structures, cartilage, labrum, rotator cuff, muscle atrophy and capsule were recorded.  *No specific classifications.* |
| **Cooper et al 2022** USA | Athletes (elite level rock climbers) | 50 volunteer elite rock climbers. Recruitment was aided by advertisements in climbing gyms in Colorado, USA, and across various online platforms, such as climbing training and route forums, as well as through social media. Climbers were eligible to participate in the study if they had been climbing at a grade of 5.11 or higher for at least 5 years; had no shoulder-related complaints; had no history of previous shoulder surgery or significant injury, including previous shoulder surgery or dislocation, rotator cuff or glenoid labrum tear, or fracture; and were willing and able to undergo MRI. | **Subacromial/subdeltoid bursitis**: Fluid distension and thickening in the bursa  **Acromioclavicular joint degeneration**: Any of the following: capsular synovial scarring, synovitis, bony ridging, irregular pitting/cystic change, edema along an articular surface, chronic appearing subluxation of either bone, thinning, fissuring, or more severe degeneration of a cartilage surface; excess joint fluid can be an associated finding but is not degeneration by itself  *No specific classifications.* |

# Supplementary Table 9 – The certainty of evidence

| **Grading of the level of evidence.** | | | | | | | |
| --- | --- | --- | --- | --- | --- | --- | --- |
| **No of shoulders (No of studies)** | **Study population** | **Risk of bias** | **Inconsistency** | **Imprecision** | **Indirectness** | **Publication bias** | **Overall certainty of evidence** |
| **AC OA** | | | | | | | |
| 20 (1) | Population-based | Serious | Not serious | Very serious | Serious | Not serious | Very low |
| 1443 (13) | Miscellaneous | Serious | Very serious | Serious | Serious | Not serious | Very low |
| 351 (9) | Athletes | Serious | Very serious | Serious | Serious | Not serious | Very low |
| **SA bursa abnormality** | | | | | | | |
| 20 (1) | Population-based | Serious | Not serious | Very serious | Serious | Not serious | Very low |
| 1538 (14) | Miscellaneous | Serious | Serious | Serious | Serious | Not serious | Very low |
| 533 (9) | Athletes | Serious | Very serious | Serious | Serious | Not serious | Very low |
| **SA space abnormality** | | | | | | | |
| 20 (1) | Population-based | Serious | Not serious | Very serious | Serious | Not serious | Very low |
| 1205 (9) | Miscellaneous | Serious | Serious | Serious | Serious | Not serious | Very low |
| 28 (1) | Athletes | Serious | Not serious | Very serious | Serious | Not serious | Very low |
| **SA calcification** | | | | | | | |
| 20 (1) | Population-based | Serious | Not serious | Very serious | Serious | Not serious | Very low |
| 1487 (8) | Miscellaneous | Serious | Not serious | Serious | Serious | Not serious | Very low |
| 285 (4) | Athletes | Serious | Serious | Serious | Serious | Not serious | Very low |

# Supplementary Table 10 – Comparison of asymptomatic and symptomatic shoulders

**Asymptomatic**

**Symptomatic**

|  | Prevalence of glenohumeral (GH) joint osteoarthritis (OA), humeral head cysts, labrum abnormalities, and long head of biceps (LHB) abnormalities in asymptomatic and symptomatic shoulders | | | | | | | |
| --- | --- | --- | --- | --- | --- | --- | --- | --- |
| **Study** | **Study population** | **Age, years**  **(range)** | **Women (%)** | **Number of shoulders** | **AC-joint OA*,  % (n/N)** | **SA bursa abnormality†, % (n/N)** | **SA space abnormality‡, % (n/N)** | **SA calcification,  % (n/N)** |
| **X-ray studies** | | | | | | | | |
| **Gill et al 2014** | Population-based¶ | 64.8 (56–74) | 60 | 20 | 95 (19/20) | NR | 20 (4/20) | 5 (1/20) |
| **Gill et al 2014** | Population-based¶ | 64.8 (56–74) | 60 | 10 | 100 (10/10) | NR | 30 (3/10) | 20 (2/10) |
| **Khoschnau et al 2020** | Miscellaneous§ (healthcare population and volunteers) | 66 (50–75) | 51 | 129 | 31 (40/129) | NR | NR | NR |
| **Khoschnau et al 2020** | Miscellaneous§ (healthcare population and volunteers) | 66 (50–75) | 51 | 83 | 33.7 (28/83) | NR | NR | NR |
| **US studies** | | | | | | | | |
| **Sansone et al 2016**  Italy | Miscellaneous§ (healthcare population) | 38.5 (18–60) | 100 | 509 | NR | NR | NR | 13.6 (69/509) |
| **Sansone et al 2016**  Italy | Miscellaneous§ (healthcare population) | 38.5 (18–60) | 100 | 95 | NR | NR | NR | 35.8 (34/95) |
| **Eliason et al 2022** | Miscellaneous# (healthcare population) | 45.0 (20–59)  (20–29) (30–39) (40–49) (50–59) | 53 | 115  14  19  35  47 | 13 (15/115)  0 (0/14)  21.1 (4/19)  17.1 (6/35)  10.6 (5/47) | 73 (84/115)  35.7 (5/14)  89.5 (17/19)  71.4 (25/35)  78.7 (37/47) | NR | 17.4 (20/115)  0 (0/14)  10.5 (2/19)  20 (7/35)  23.4 (11/47) |
| **Eliason et al 2022** | Miscellaneous# (healthcare population) | 45.0 (20–59)  (20–29) (30–39) (40–49) (50–59) | 53 | 115  14  19  35  47 | 20 (23/115)  0 (0/14)  21.1 (4/19)  25.7 (9/35)  21.3 (10/47) | 88.7 (102/115)  57.1 (8/14)  100 (19/19)  85.7 (30/35)  95.7 (45/47) | NR | 25.2 (29/115)  7.1 (1/14)  26.3 (5/19)  22.9 (8/35)  31.9 (15/47) |
| **Brasseur et al 2004** | Athletes¶  (veteran Tennis players) | 55 (37–77) | 43.3 | 119 | NR | 22.7 (27/119) | NR | 25.2 (30/119) |
| **Brasseur et al 2004** | Athletes¶  (veteran Tennis players) | 55 (37–77) | 43.3 | 31 | NR | 12.9 (4/31) | NR | 38.7 (12/31) |
| **Suzuki et al 2021** | Athletes§  (masters level swimmers) | 51.8 (33–65) | 65 | 60 | NR | 11.7 (7/60) | NR | 16.7 (10/60) |
| **Suzuki et al 2021** | Athletes§  (masters level swimmers) | 51.4 (33–65) | 60 | 20 | NR | 10 (2/20) | NR | 35 (7/20) |
| **MRI studies** | | | | | | | | |
| **Gill et al 2014** | Population-based¶ | 64.8 (56–74) | 60 | 20 | 85 (17/20) | 90 (18/20) | NR | NR |
| **Gill et al 2014** | Population-based¶ | 64.8 (56–74) | 60 | 10 | 100 (10/10) | 100 (10/10) | NR | NR |
| **Chandnani et al 1992** | Miscellaneous¶  (volunteers) | (25–55) | NR | 20 | 35 (7/20) | 0 (0/20) | NR | NR |
| **Chandnani et al 1992** | Miscellaneous¶ (volunteers) | (25–55) | NR | 20 | 55 (11/20) | 30 (6/20) | NR | NR |
| **Barreto et al 2019** | Miscellaneous#  (healthcare population) | 39.4 (18–77) | 46.3 | 123 | 73.2 (90/123) | 52.8 (65/123) | 13 (16/123) | NR |
| **Barreto et al 2019** | Miscellaneous#  (healthcare population) | 39.4 (18–77) | 46.3 | 123 | 79.7 (98/123) | 60.1 (75/123) | 15.5 (19/123) | NR |
| **Reuter et al 2008** | Athletes¶  (ironman participants) | 35 (29–62) | 28.6 | 7 | 71.4 (5/7) | NR | NR | NR |
| **Reuter et al 2008** | Athletes¶  (ironman participants) | 39 (27–59) | 28.6 | 16 | 62.5 (10/16) | NR | NR | NR |
| **Celliers et al 2017** | Athletes§  (elite swimmers) | 18.9 (16–25) | 45 | 29 | 34.5 (10/29) | 34.5 (10/29) | NR | NR |
| **Celliers et al 2017** | Athletes§  (elite swimmers) | 18.9 (16–25) | 45 | 11 | 36.4 (4/11) | 45.5 (5/11) | NR | NR |

* = Osteophytes, joint effusion, bone oedema, bony ridging, elevated bone marrow signal, joint narrowing, joint degeneration, joint hypertrophy, articular surface irregularity, articular cartilage thinning, fissuring or degeneration, cortical irregularities, margin irregularity, bone sclerosis, erosions, osteoarthritis, synovial scarring, cystic change

† = Bursal effusion, bursal thickening, bursal hypertrophy

‡ = SA space narrowing, SA spurs, SA enthesophytes, acromion osteophytes, acromiohumeral distance (abnormal/narrow), Type III acromion (hooked), SA impingement, AC joint osteophytes impinging the supraspinatus tendon

§ = Not specified whether a) asymptomatic and symptomatic shoulders within the same individuals or b) asymptomatic and symptomatic shoulders from different individuals within the same study population were compared

¶ = Asymptomatic and symptomatic shoulders from different individuals within the same study population

# = Asymptomatic and symptomatic shoulders within the same individuals

Supplementary Figure 1 – Scatter plots of athletes vs. non-athletes

Scatter plots showing the prevalence according to age in athletes and non-athlete populations. Although there was a large variation in prevalence rates, the overall trend showed that athlete populations were either younger and/or had higher prevalence rates than non-athlete populations of the same age.
